# Sequential Deoxygenation of CO_2_ and NO_2_^–^ via Redox-Control of a Pyridinediimine Ligand with a Hemilabile Phosphine

**DOI:** 10.1021/acs.inorgchem.3c02323

**Published:** 2023-09-05

**Authors:** Hanalei R. Lewine, Allison G. Teigen, April M. Trausch, Kaitlyn M. Lindblom, Takele Seda, Eric W. Reinheimer, Tim Kowalczyk, John D. Gilbertson

**Affiliations:** † Department of Chemistry, Western Washington University, Bellingham, Washington98225, United States; ‡ Department of Physics, Western Washington University, Bellingham, Washington98225, United States; § Rigaku Oxford Diffraction, Woodlands, Texas77381, United States

## Abstract

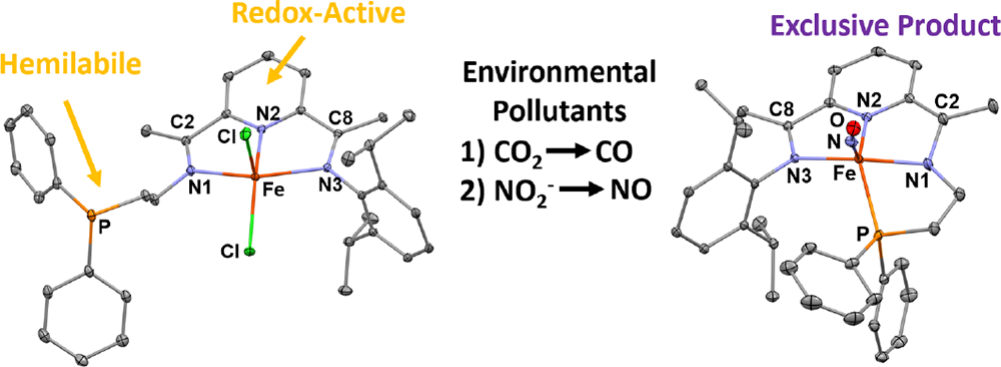

The deoxygenation of environmental pollutants CO_2_ and NO_2_^–^ to form value-added products is reported. CO_2_ reduction with subsequent CO release and NO_2_^–^ conversion to NO are achieved via the starting complex Fe(^PPh^PDI)Cl_2_ (**1**). **1** contains the redox-active pyridinediimine (PDI) ligand with a hemilabile phosphine located in the secondary coordination sphere. **1** was reduced with SmI_2_ under a CO_2_ atmosphere to form the direduced monocarbonyl Fe(^PPh^PDI)(CO) (**2**). Subsequent CO release was achieved via oxidation of **2** using the NO_*x*_^–^ source, NO_2_^–^. The resulting [Fe(^PPh^PDI)(NO)]^+^ (**3**) mononitrosyl iron complex (MNIC) is formed as the exclusive reduction product due to the hemilabile phosphine. **3** was investigated computationally to be characterized as {FeNO}^7^, an unusual intermediate-spin Fe(III) coupled to triplet NO^–^ and a singly reduced PDI ligand.

## Abstract 2

Utilizing ligand hemilability and redox-activity, the deoxygenation of the environmental pollutants CO_2_ and NO_2_^−^ to form value-added products is reported. CO_2_ reduction with subsequent CO release and NO_2_^−^ conversion to NO is reported. The hemilabile phosphine allows for the selective formation of the {FeNO}^7^ mononitrosyl iron complex.

## Introduction

Highly oxygenated species play a vital role in global biogeochemical cycles such as the carbon cycle (carbon dioxide, CO_2_) and the nitrogen cycle (nitrate/nitrite, NO_3_^–^/NO_2_^–^). Buildup of these polyoxygenated species makes them recalcitrant environmental pollutants given their thermodynamic and kinetic stability. For example, NO_2_^–^ is a pervasive pollutant in groundwater and poses a serious threat to human health.^1,2^ Groundwater treatment is an extremely difficult task,^3^ making remediation of the recalcitrant contaminant an area of great interest. Typical strategies for O atom extraction from nitrogen oxyanions via the Mashima^4^ or borylating^5^ reagents, as well as through oxygen-deficient polyoxo-clusters,^6^ have recently been shown to be viable for selective deoxygenation of NO_*x*_^–^. Several NO_*x*_^–^ conversions have been reported utilizing transition metal complexes;^7−16^ however, few are reported to produce value-added chemicals.^17−19^

CO_2_ is a significant environmental contaminant as well. Sensible methods of CO_2_ utilization are needed for its mitigation as a greenhouse gas.^20,21^ Carbon monoxide (CO) is a valuable feedstock produced industrially by steam reforming fossil fuels to produce syngas and is a versatile chemical precursor and fuel.^22−24^ The production of value-added CO from the waste product CO_2_ is an attractive route to the production of C_1_ source(s).^25^ Iron-mediated CO_2_ deoxygenation to CO typically requires the presence of an O atom acceptor (or a second molecule of CO_2_).^26−31^ The subsequent inert Fe–CO bonds that are formed prevent the release of CO in most systems. One way to overcome this barrier is to use photochemical or electrochemical methods.^32,33^ We previously reported the conversion of CO_2_ to CO on iron utilizing the redox-active pyridinediimine (PDI) ligand scaffold, utilizing the ligand oxidation state as a viable redox-switch for CO release.^34^

In addition, the PDI scaffold can be used in the ligand-based reduction of NO_2_^–^ to NO on the dicarbonyl complex [Fe(H^DEA^PDI)(CO)_2_]^+^ (where ^DEA^PDI = [(2,6-^i^PrC_6_H_3_)(N = CMe)(N(Et)_2_C_2_H_4_)(N = CMe)C_5_H_3_N]). The reaction forms the dinitrosyl iron complex (DNIC) [Fe(^DEA^PDI)(NO)_2_]^+^ as the sole nitrogen containing product.^35^ DNICs are notoriously inert,^36,37^ as illustrated by the sheer number reported in the literature,^38,39^ and [Fe(^DEA^PDI)(NO)_2_]^+^ is no exception. Given the thermodynamic stability of DNICs, it is no surprise that NO_*x*_^–^ reduction on iron complexes would yield stable complexes. Unfortunately, the stability of the DNIC hinders our efforts to utilize iron to further reduce the NO_*x*_^–^ derived products to form more inert (N_2_) or possibly value-added (NH_3_) products. A more plausible route would be to go through a mononitrosyl iron complex (MNIC), which are key intermediates in numerous NO_*x*_^–^ reduction schemes.^40−42^ Selectivity of MNIC over DNIC formation is a crucial problem in iron-mediated NO_*x*_^–^ reduction.^43^

**Scheme 1:**
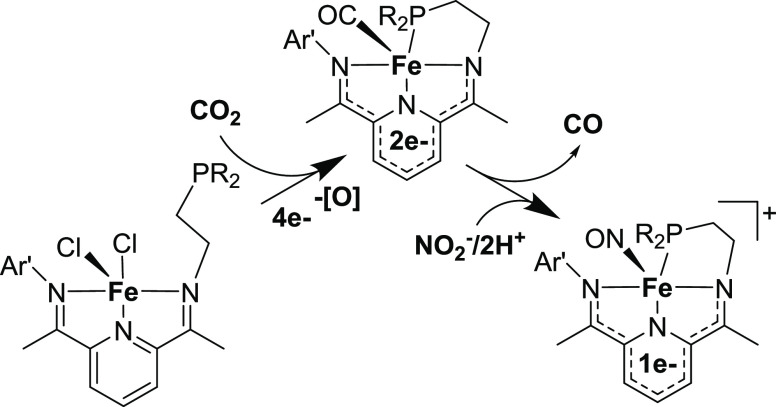
Reaction Scheme Outlining CO_2_ Reduction to Form CO and Subsequent CO Release/Reduction of NO_2_^–^ on Hemilabile FePDI Complex

The work reported here (Scheme 1) exploits redox switching of the PDI scaffold and pendant group hemilability for the reduction of NO_2_^–^ to NO. The Fe(^PPh^PDI)(CO) species active for NO_2_^–^ reduction can be derived from a waste product (CO_2_) to produce value-added CO (which is released in the subsequent NO_2_^–^ deoxygenation step). The hemilabile phosphine avoids the formation of the DNIC, producing the [Fe(^PPh^PDI)(NO)]^+^ MNIC exclusively.

## Results and Discussion

**Figure 1:**
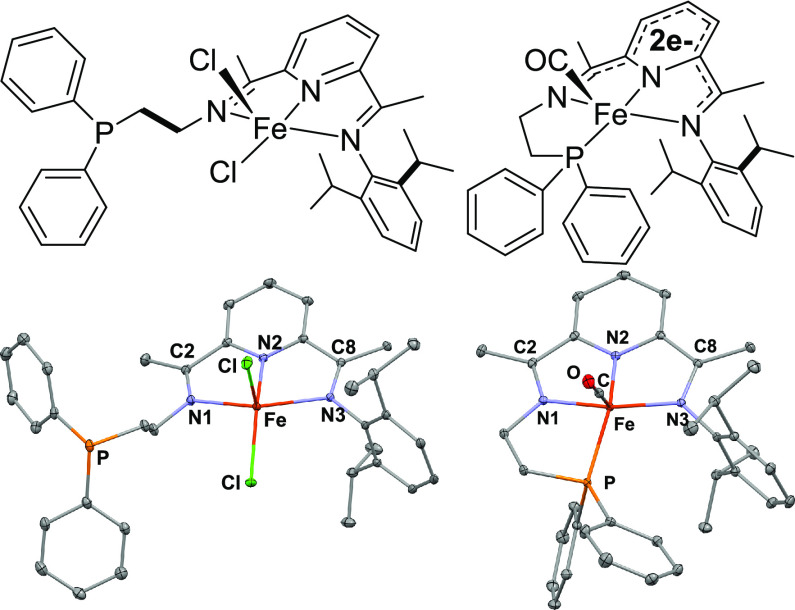
Chemdraw (top) and solid state structure (bottom) of Fe(^PPh^PDI)Cl_2_ (**1**, left) and Fe(^PPh^PDI)(CO) (**2**, right). Selected bond lengths (Å) and angles (deg) for Fe(^PPh^PDI)Cl_2_ (1): Fe(1)–N(1) 2.217(1), Fe(1)–N(2) 2.0084(9), Fe(1)–N(3) 2.240(1), Fe(1)–Cl(1) 2.299(4), Fe(1)–Cl(2) 2.316(5), N(1)–C(2) 1.286(1), N(3)–C(8) 1.290(1), C(2)–C(3) 1.487(2), C(7)–C(8) 1.486(2), and N(1)–Fe(1)–N(3) 142.88(4), N(2)–Fe(1)–Cl(1) 141.39(3). Selected bond lengths (Å) and angles (deg) for Fe(^PPh^PDI)(CO) (2): Fe(1)–N(1) 1.964(9), Fe(1)–N(2) 1.838(1), Fe(1)–N(3) 1.924(1), Fe(1)–C(36) 1.761(1), Fe(1)–P(1) 2.2023(4), N(1)–C(2) 1.344(1), N(3)–C(8) 1.332(2), C(2)–C(3) 1.423(2), C(7)–C(8) 1.427(1), and N(1)–Fe(1)–N(3) 156.83(4), N(2)–Fe(1)–P(1) 148.67(3).

The complex Fe(^PPh^PDI)Cl_2_ (**1**) was synthesized from the Fe-mediated Schiff base condensation of [(2,6-^i^PrC_6_H_3_N=CMe)(O=CMe)C_5_H_3_N] with 2-(diphenylphosphino)ethylamine in the presence of FeCl_2_ in EtOH in 75% yield (see SI for details). Single crystals of the blue product were obtained from the layering of diethyl ether onto a concentrated solution of CH_2_Cl_2_. An ORTEP view of **1** is shown in Figure 1 (left). The iron center is five-coordinate with a square-pyramidal geometry (τ = 0.02).^44^ The nitrogen atoms of the PDI ring along with one chlorine atom make up the basal plane with the other chlorine atom occupying the apical position. The solid-state structure also reveals that the pendant diphenylphoshine group is not bound to the iron center. The bond lengths and angles are similar to those of other structurally characterized high-spin square-pyramidal iron complexes containing PDI ligands.^45−47^ The C_imine_–N_imine_ (1.286(1) and 1.290(1) Å) and C_imine_–C_ipso_ (1.487(2) and 1.486(2) Å) bonds in **1** are consistent with a neutral ^PPh^PDI core. The zero-field Mössbauer parameters of **1** confirm the assignment of a high-spin Fe(II) center [δ = 0.859(2); Δ*E*_Q_ = 0.979(4) mm/s].^45,48,49^

We have shown with numerous types of Fe(PDI)X_2_ complexes (where X = Cl^–^ or Br^–^) that reduction with NaHg under a CO atmosphere produces the direduced dicarbonyl Fe(PDI)(CO)_2_ species.^50,51^ In the case of **1**, however, NaHg reduction under CO results in a mixture of direduced monocarbonyl and direduced dicarbonyl species (see SI for details). Given the difficulties in separation of the two species, SmI_2_ in THF was used as the reductant instead, yielding the direduced monocarbonyl Fe(^PPh^PDI)(CO) (**2**) exclusively when one equivalent of CO was used in the reaction. Slow evaporation of a saturated diethyl ether solution of **2** yielded a red, diamagnetic, crystalline solid in 70% yield. The ATR-FTIR spectrum of **2** displays one ν_CO_ stretch at 1853 cm^–1^. An ORTEP view of **2** is shown in Figure 1 (right). The Fe center is five-coordinate with a square-pyramidal geometry (τ = 0.14). The C_imine_–N_imine_ bond lengths in **2** are elongated to 1.332(2) and 1.344(1) Å, and the C_imine_–C_ipso_ bond lengths are contracted to 1.427(1) and 1.423(2) Å, indicative of a direduced species. The room temperature zero-field Mössbauer parameters (SI) [δ = 0.213(1); Δ*E*_Q_ = 0.704(2) mm/s] support the assignment of an *S* = 0 iron center with a doubly reduced ^PPh^PDI ligand. The isomer shift in **2** is larger than that of the dicarbonyl systems, given the presence of one π-backbonding CO ligand instead of two.^52^

The hemilabile diphenylphosphino group^53^ is bound to the iron center in **2**. The Fe(1)–P(1) bond distance of 2.2023(4) Å forms a κ-4 N,N,N,P chelate in the basal plane of the molecule with the CO ligand occupying the apical position. The ^31^P{^1^H} NMR spectrum of diamagnetic **2** in CD_2_Cl_2_ confirms that the diphenylphosphino arm is bound to the iron center in solution as well, appearing at 64 ppm. This value is shifted well downfield of the free arm, which appears at −21 ppm, due to the deshielding that occurs upon metal binding.

**Figure 2:**
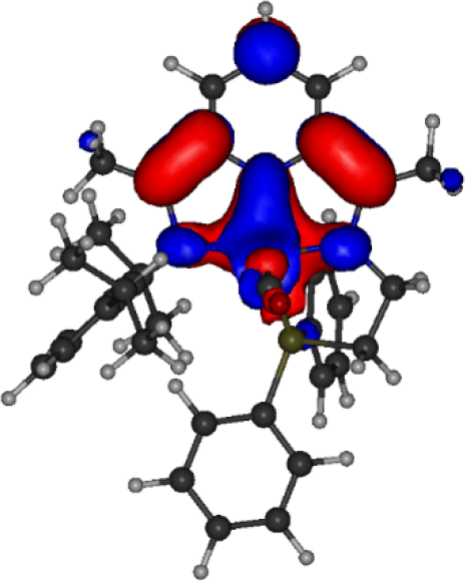
HOMO of direduced monocarbonyl Fe(^PPh^PDI)(CO) (**2**).

The electronic structure of **2** was computed, given the unusual character of **2** as a direduced, monocarbonyl complex. Previous analyses of the closed-shell frontier orbitals of direduced, dicarbonyl Fe(PDI)(CO)_2_ complexes suggest that they are best represented as a resonance hybrid of a PDI^0^ ligand on a Fe(0) d^8^ center and a PDI^2–^ ligand on a low-spin Fe(II) center.^49,54^ We computed the closed-shell wave function for **2**, and the resulting electronic character is consistent with the previous studies on the dicarbonyl systems. The HOMO, shown in Figure 2, is distributed across both the iron center and the PDI ligand, reinforcing the notion that the ground-state electronic structure is a hybrid of Fe(0) and Fe(II) with significant metal–ligand covalent character.

Complex **2** can also be synthesized with CO_2_ as the source of the CO ligand and SmI_2_ as the O atom acceptor (eq 1). When the reduction of **1** is repeated with CO_2_ in place of CO, complex **2** is formed in 22% yield (see SI for details). The yield of **2** is lower in the CO_2_ reaction due to the requisite O-atom accounting,^34^ and the four electrons required (two electrons to reduce the PDI ligand, and two electrons to reduce CO_2_ to CO). If the stoichiometry is adjusted to account for four equiv of electrons (SmI_2_), **2** is formed in similar yield (71%) from CO_2_ as from CO in the reaction described above. Control reactions of 0.1 M solutions of SmI_2_ in THF and CO_2_ resulted in no reaction. These observations show that value-added CO can be generated from CO_2_ with **1** and SmI_2_.


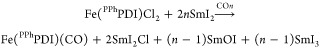
1

**Figure 3:**
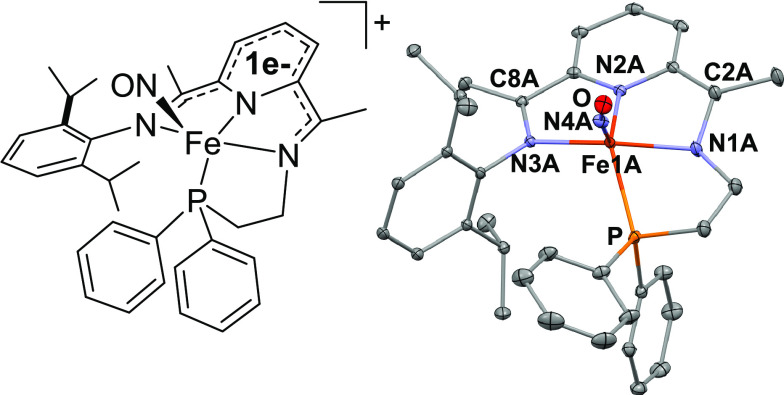
Chemdraw (left) and solid state structure (right) of [Fe(^PPh^PDI)(NO)]^+^ (**3**). The counterion was omitted for clarity. Selected bond lengths (Å) and angles (deg): Fe(1A)–N(1A) 1.926(2), Fe(1A)–N(2A) 1.880(2), Fe(1A)–N(3A) 2.029(2), Fe(1A)–N(4A) 1.669(2), Fe(1A)-P(1A) 2.2970(5), N(1A)–C(2A) 1.315(3), N(3A)–C(8A) 1.317(3), C(2A)-C(3A) 1.430(3), C(7A)-C(8A) 1.440(3), and N(1A)–Fe(1A)-N(3A) 152.91(7), N(2A)–Fe(1A)-P(1A) 140.35(6), Fe(1A)-N(4A)-O(1A) 164.2(2).

Complex **2** appears to be similar to our previously reported complexes with a hemilabile PDI ligand, where a pendant amine was capable of varying denticity to form the κ-4 binding mode of the pincer. This structural change was responsible for the stabilization of unusual {FeNO}^7^ mononitrosyl intermediates in the NO_2_^–^ reduction reaction, ultimately forming stable {Fe(NO)_2_}^9^ DNICs.^50^ Given the formation of the monocarbonyl (not observed with the hemilabile amines) and the stronger field phosphine group, we reasoned that **2** would also react to stabilize a {FeNO}^7^ mononitrosyl complex and avoid the formation of the DNIC. Reaction of **2** with either two equiv of [HNEt_3_][BPh_4_] or [HNEt_3_][PF_6_] and one equivalent of NaNO_2_ in THF/MeOH caused a color change from red to brown/green. After purification (see SI for details), X-ray-quality crystals of the mononitrosyl [Fe(^PPh^PDI)(NO)][PF_6_] (**3**) were obtained from the layering of either diethyl ether or pentane onto a concentrated solution of **3** in CH_2_Cl_2_ (98% yield). **3** crystallizes in the triclinic P-1 space group and contains two independent molecules per asymmetric unit. An ORTEP view of one of the independent molecules of **3** is shown in Figure 3 (right). The Fe center is five-coordinate with a distorted square-pyramidal geometry (τ = 0.21), and the phosphine is still part of the primary coordination sphere of the iron center. The PDI backbone is in the monoreduced form as indicated by the C_imine_–N_imine_ bond lengths of 1.315(2) and 1.317(3) Å and the C_imine_–C_ipso_ bond lengths 1.430(3) and 1.440(3) Å. The Fe1A-N4A-O1A bond angle is 164.2(2)° and the Fe–N(O) bond length in **3** is 1.669(2) Å, while the N–O bond length is 1.175(3) Å. The complex is diamagnetic in the solid state and solution. The ATR-FTIR spectrum of **3** displays one ν_NO_ stretch at 1708 cm^–1^, which is shifted to 1675 cm^–1^ when Na^15^NO_2_ was used in the synthesis. The room temperature zero-field Mössbauer parameters are δ = 0.127(2) mm/s; Δ*E*_Q_ = 1.012(6) mm/s. Taken in conjunction with the structural data, an Enemark–Feltham^55^ assignment of {FeNO}^7^ is appropriate. The ^31^P{^1^H} NMR spectrum of diamagnetic **3** in CD_2_Cl_2_ confirms that the diphenylphosphino arm is bound to the iron center in solution. The resonance is shifted upfield to 50 ppm, consistent with the observed decrease in the isomer shift upon changing from a CO to a NO ligand.
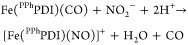
2

**Figure 4:**
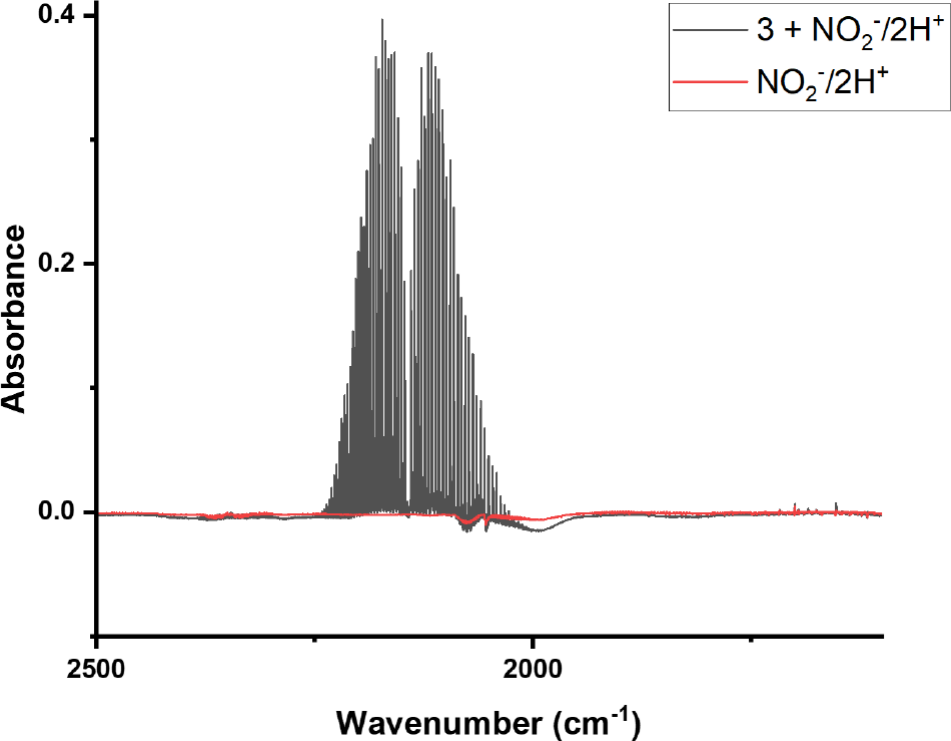
Gas phase FTIR of the reaction of **3** with one equiv of NO_2_^–^ and two equiv of H^+^ (black trace) demonstrating CO release with no gaseous N-oxides or CO_2_. The red trace is the same reaction without **3** present.

As shown in eq 2, reduction of NO_2_^–^ results in the release of the CO_2_-derived CO ligand.^56^ Inspection of the headspace above the reaction of monocarbonyl **2** with NO_2_^–^/2H^+^ reveals the presence of CO. As shown in the black trace in Figure 4, only liberated CO is observed in the headspace. Gaseous N-oxides such as NO and N_2_O were not detected. CO_2_ was also absent, demonstrating that CO is not acting as the reductant/oxygen atom acceptor in the reaction.^8,57^ The control reaction with NO_2_^–^ and 2H^+^ without **3** does not produce N-oxides either, as shown in the red trace in Figure 4.

To understand the impact of replacing a hemilabile amine with phosphine on the electronic structure of these Fe(NO)(PDI) complexes, we characterized **3** computationally via broken-symmetry density functional theory (BS-DFT) geometry optimizations at the PBE0/def2-TZVP(-f) level^58^ with the RIJCOSX approximation^59^ in ORCA 4.0.1.2.^60^ The lowest-energy electronic configuration for **3** is the BS(*2,2*) state, which lies below the UKS ground state by 5.5 kcal/mol.^61^ The triplet ground state and BS(*1,1*) state lie further above the BS(*2,2*) state by 12.2 and 14.0 kcal/mol, respectively. We also computed the lowest energy ground state for [Fe(^Py^PDI)(NO)]^+^ (**4**) (a hemilabile MNIC, with pyridine as the amine-donating group) and previously published [Fe(^Pyrr^PDI)(NO)]^+^ (**1a**) (pyrrolidine as the amine-donating group).^50^ The lowest-energy state for **4** is, likewise, the BS(*2,2*) state. Key geometric properties of the optimized structures are shown in Table S1. The optimized bond lengths and FeNO angle of **4** resemble those of **1a**, with a slightly more acute FeNO angle and are consistent with a neutral radical state for NO in this complex. The FeNO angle for the BS(*2,2*)-optimized structure of **3** (162.2°) exceeds that of **4** and **1a** (147.5°) but is not quite as linear as the angle measured for **3** by X-ray crystallography (164.2°).

Frontier corresponding orbital overlaps of **3** and **4** are helpful for visualizing the extent of symmetry breaking in the BS wave functions^62^ (Figures S15 and S16). In both cases, the dominant qualitative difference between the highest-occupied spin-up and spin-down corresponding orbitals in both complexes is the extent to which each corresponding orbital extends over the PDI ligand, indicating radical character for this ligand in both structures. The overlap between the highest occupied corresponding orbitals of **3** is S_αβ_ = 0.713, quite close to the overlap observed for **1a** (S_αβ_ = 0.729)^50^ but significantly larger than the overlap S_αβ_ = 0.645 observed for **4**. These results support the assignment of radical anion character to the oxidation state of PDI in **3** and **4**. However, they do not explain the difference in the FeNO angles of the two complexes. For that, we turned to a more local probe of the electronic structure.

**Figure 5:**
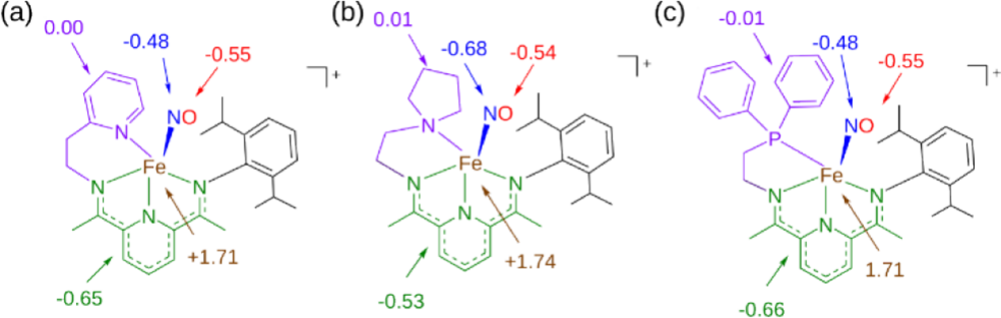
NBO spin populations of (a) **4**, (b) **1a**, and (c) **3** at the PBE0/LANL2DZ level of theory.

To better understand the electronic character locally around the redox-active ligands, we performed natural bond orbital (NBO) analysis on the broken-symmetry ground states of **3** and **4** at the PBE0/LANL2DZ level in Q-Chem 5.0.^6363^ Natural spin populations are illustrated for the metal center, redox-active ligands, and hemilabile ligands in Figure 5. For **4**, NBO spin populations on Fe (+1.71), NO (−1.03), and PDI (−0.65) in the BS(2,2) solution suggest {FeNO}^7^ character like that of **1a** with a singly reduced PDI ligand, Fe^3+^(↑↑↑)NO^–^(↓↓)PDI^•–^(↓), with the excess spin on PDI more delocalized due to conjugation. For **3**, the natural spin densities on the Fe center, NO ligand, and nonhydrogen PDI atoms are nearly identical at +1.71, −1.13, and −0.66, respectively. The smaller extent of spin polarization on NO observed in **4** and **3** relative to **1a** suggests the possibility of a minor resonance contribution to **4** and **3** from a configuration in which the NO has neutral radical character; however, NBO analysis places partial negative charges of similar magnitude, between −0.2 and −0.3, on both the NO and PDI units of **2** and **3**. Therefore, any resonance contribution with an asymmetric charge density on NO relative to PDI would need to be counterbalanced by a resonance contribution with the opposing effect. Like the pyrrolidine in **1a** and the pyridine in **4**, the phosphine ligand in **3** bears negligible net spin density: the NBO spin density on the phosphorus atom itself is negligible (<0.01), demonstrating that this ligand does not contribute meaningfully to the open-shell character of the complex and that the substitution of N for P on the hemilabile ligand does not fundamentally change the spin character of the Fe center.

With the MNIC synthesis from NO_2_^–^ established, we tested the propensity of complex **3** to react further to form the DNIC. When the NO_2_^–^ reduction reaction with **2** is repeated with two equiv of NaNO_2_ and four H^+^ equiv, only MNIC formation is observed. Alternatively, when **3** is mixed with a second equivalent of NO_2_^–^ and acid, no reaction is observed, even upon heating to 80 °C overnight. These observations make it clear that the MNIC formation is exclusive to DNIC formation for the phosphine donor. This reactivity is the reverse of what is observed when N-donor amines are used. These results can be explained, in part, by the donor–acceptor abilities of the differing hemilabile groups. The N-donating pyrrolidine arm (when bound to the metal) is a σ-donor, while the diphenylphosphino group is more of a π-acid. The Fe-NO angle in **3** reflects this, as it is much more linear (164°) than in **1a** (151°). In addition, the ν_NO_ stretch in **3** (1708 cm^–1^) is shifted to a higher wavenumber from **1a** (1667 cm^–1^), illustrating the better π-accepting abilities of the phosphine compared to the pyrrolidine group. Lastly, the π-acceptor character of the diphenylphosphino group is corroborated computationally with an NBO charge of +0.52 on the phosphorus versus −0.25 for the pyrrolidine nitrogen of [Fe(^Pyrr^PDI)(NO)]^+^. Consistent with the resistance of **3** to form the DNIC, the Fe–P Wiberg bond index of 0.40 is stronger than the Fe–N Wiberg bond index of 0.26 for the pyrrolidine arm.^63^ The stronger π-acceptor renders the iron center unable to react further with 1 equiv of NO_2_^–^, eliminating the DNIC formation.

**Table 1:** Selected Spectroscopic and Crystallographic Data for Fe(PDI) Complexes^a^

	**1**	**2**	**3**	**3**^**(2,2)**^^b^
C(2)–N(1)	1.286(1)	1.344(1)	1.315(3)	1.1367
C(8)–N(3)	1.290(1)	1.332(2)	1.317(3)	1.3028
C(2)–C(3)	1.487(2)	1.423(2)	1.430(3)	1.4276
C(7)–C(8)	1.486(2)	1.427(1)	1.440(3)	1.4469
Fe(1)–N(1)	2.217(1)	1.964(9)	1.926(2)	1.9429
Fe(1)–N(2)	2.0084(9)	1.838(1)	1.880(2)	1.8566
Fe(1)–N(3)	2.240(1)	1.924(1)	2.029(2)	2.0467
Fe(1)–P(1)		2.2023(4)	2.2970(5)	2.3635
Fe(1)–N(4)(O)			1.669(2)	1.7124
N(4)–O(1)			1.175(3)	1.1580
Fe–N(4)-O(1)			164.2(2)	162.2
ν_CO_,ν_NO_ (cm^–1^)		1853	1708	
S =	2	0	0	0
δ (mm/s)	0.859(2)	0.213	0.127(2)	
Δ*E*_Q_ (mm/s)	0.979(4)	0.704	1.012(6)	

a Bond lengths (Å) and angles (deg).

b BS(2,2) solution.

## Conclusions

In conclusion, we have presented an iron-based system active in the deoxygenation of pervasive environmental pollutants CO_2_ and NO_2_^–^ utilizing a redox-active ligand scaffold merged with a hemilabile pendant phosphine. The redox state of the ligand and the pendant group hemilability can be exploited to produce value-added CO (which is released in the subsequent NO_2_^–^ deoxygenation step). The hemilabile phosphine avoids the formation of a DNIC, forming {FeNO}^7^ MNIC **3** exclusively. **3** appears electronically equivalent to our previously reported diamagnetic {FeNO}^7^ MNICs, which are somewhat unusual in that they are intermediate spins (is).^50^ Classically, high spins (hs) {FeNO}^7^ are inert, whereas low spins (ls) {FeNO}^7^ are more reactive.^38^ In this case, is-{FeNO}^7^**3** is also inert, while is-{FeNO}^7^ complexes **4** and **1a** both react with NO_2_^–^/2H^+^ to form the corresponding DNICs. As stated above, the diphenylphosphino group in **3** is more of a π-acid and the N-donating arms in **4** and **1a** are more σ-donating, which may be affecting the selectivity of MNIC over DNIC formation. Additionally, the N-donating arms in **4** and **1a** are proton responsive. Given that the NO_2_^–^ reduction reaction requires exogenous protons, the lack of proton responsivity in **3** could also likely be preventing the complex from reacting further to form the {Fe(NO)_2_}^9^ DNIC. We are currently investigating the formation and reactivity of the {FeNO}^6/8^ analogs of **3** in order to achieve further functionalization of the NO ligand.

## Supporting Information Available

X-ray crystallographic, spectroscopic, and computational data (PDF)
