# Supplementary material for: Sequential Deoxygenation of CO2 and NO2– via Redox-Control of a Pyridinediimine Ligand with a Hemilabile Phosphine
Source: Inorg Chem. 2023 Sep 5;62(37):15173–9. doi: 10.1021/acs.inorgchem.3c02323 (PMC10520972; doi:10.1021/acs.inorgchem.3c02323)
Supplement: Supplementary file 1 — ic3c02323_si_001.pdf [file ic3c02323_si_001.pdf]

Supporting Information for

Sequential Deoxygenation of CO_2 and NO_2^- via Redox-Control of a Pyridinediimine Ligand with a Hemilabile Phosphine.

Hanalei R. Lewine,[†] Allison G. Teigen,[†] April M. Trausch,[†] Kaitlyn M. Lindblom,[†] Takele Seda,[‡] Eric W. Reinheimer,[§] Tim Kowalczyk,[†] and John D. Gilbertson[*,†]

[†]Department of Chemistry, Western Washington University, Bellingham, Washington 98225, United States
[‡]Department of Physics, Western Washington University, Bellingham, Washington 98225, United States
[§]Rigaku Oxford Diffraction, Woodlands, TX 77381, USA
*gilberj4@wwu.edu

Table of Contents

Methods	S2-S3
Experimental	S4-S6
$Fe(^{PPh}PDI)Cl_2$ (**1**)	
FT-IR	S7
Mössbauer	S7
^1H NMR	S8
$Fe(^{PPh}PDI)(CO)$ (**2**)	
FT-IR	S8
^1H NMR	S9
^{13}C NMR	S9
^{31}P NMR	S10
Mössbauer	S10
$Fe(^{PPh}PDI)(CO)$ (**2**) and $Fe(^{PPh}PDI)(CO)_2$ Mixture	
FT-IR	S11
$[Fe(^{PPh}PDI)(NO)][BPh_4]$ (**3**)	
FT-IR	S11
^1H NMR	S12
^{31}P NMR	S12
^{13}C NMR	S13
Mössbauer	S13
**Table S1**. Geometric Parameters in Optimized Geometries of MNIC structures	S14
BS(2,2) Frontier Corresponding Orbitals of **3**	S15
BS(2,2) Frontier Corresponding Orbitals of **4**	S16
Optimized Geometries of **3**, **4**, and **1a**	S17-S21
**Table S2.** Crystal Data and Refinement	S22
**Table S3.** NBO Charges and Widberg bond indices for **1a**, **3**, and **4**	S22
References	S23

Methods:
Unless otherwise noted, all reagents were purchased from commercial sources and used without further purification. Dry and air-free solvents were obtained with a PureSolv solvent still (Vacuum Atmospheres Inc.). Gases were purchased from AirGas Inc. All air-sensitive reagents were handled in an N_2 filled inert-atmosphere glovebox or on a Schlenk line following standard air-free techniques. The asymmetric ligand $[(ArN{=}C(CH_3))C_2H_3N((CH_3)C{=}O]$ was synthesized according to literature procedure.[1]

FT-IR. Fourier transform infrared spectra were collected on a Thermo iS10 FTIR spectrometer. Solution-phase IR spectra were collected in a liquid IR cell equipped with CaF_2 windows at 2 cm^{-1} resolution. Gas-phase IR spectra were collected with a Pike Technologies short-path-length (100 mm) gas transmission cell fitted with CaF_2 windows, and the resolution was set to 0.5 cm^{-1} and 32 scans. Attenuated total reflectance (ATR) IR spectra were collected on a single-bounce diamond ATR accessory at a 4 cm^{-1} resolution and 16 scans.

NMR. All NMR spectra were collected on a Bruker Advance III 500 MHz instrument operating at 499.75 MHz for ^1H spectra, 125.66 MHz for $^{13}C\{^1H\}$ spectra, and 202.40 MHz for $^{31}P\{^1H\}$ spectra. Spectra were referenced to the solvent CD_2Cl_2 for all ^1H (δ: 5.32 ppm) and $^{13}C\{^1H\}$ (δ: 53.42 ppm) experiments. Spectra were referenced to the standard D_3PO_4 in D_2O at 0 ppm for $^{31}P\{^1H\}$ experiments.

Mössbauer. Mössbauer spectra were collected on a constant-acceleration spectrometer (WissEI Gmbh, Germany) in a horizontal transmission mode using a 50 mCi ^{57}Co source. For room temperature measurements, approximately 80 mg of a sample was loaded into an acrylic sample holder and covered with Paratone-N oil to prevent oxidation. Data collection was between 2 and 7 days to get a statistically significant spectrum. Isomer shifts were normalized to that of metallic iron, and spectra were fitted assuming Lorentzian line shapes using the *NORMOS* (WissEI Gmbh) least-squares fitting program.

Evan's Balance Magnetic Susceptibility. Susceptibilities were recorded on a Johnson Matthey MSB-1 magnetic susceptibility balance that was calibrated with $Hg[Co(SCN)_4]$. Diamagnetic correction factors were calculated from Pascal's constants.[2]

X-ray Crystallography. X-ray diffraction data on single blue plate-like crystals of $Fe(^{PPh}PDI)Cl_2$ (**1**) with dimensions 0.43 x 0.19 x 0.11 m^3 as well as a brown prism of $Fe(^{PPh}PDI)(CO)$ (**2**) having dimensions 0.33 x 0.28 x 0.19 m^3 were completed after securing each to Mitegen mounts using Paratone oil and mounted on a Rigaku Oxford Diffraction (ROD) XtaLABPRO equipped with a Pilatus P200K hybrid photon counting (HPC) detector and fine-focused Mo $K_{\alpha 1}$ radiation (= 0.71073 Å). Reflection data were collected at 100 K with data collection strategies to ensure completeness and desired redundancy determined using CrysAlis^{Pro}.[3,4] Data processing for all samples was done using CrysAlis^{Pro} and included analytical absorption corrections after face indexing using the SCALE3 ABSPACK scaling algorithm.[5] The structures were solved via intrinsic phasing methods using ShelXT[6] and subjected to a least-squares refinements with ShelXL[7] within the Olex2 graphical user interface.[8] The final structural refinements included anisotropic temperature factors on all constituent non-hydrogen atoms. Hydrogen atoms in **1** were attached via the riding model at calculated positions using suitable HFIX commands. Within the structural model of **2**, peaks indicative of hydrogen atoms were observed in the difference map and freely-refined. Space groups were unambiguously verified by PLATON.[9]

X-ray diffraction data for green $[Fe(^{PPh}PDI)(NO)][PF_6]$ (**3**) were collected at 100 K on a Bruker D8 Venture using MoKα (λ = 0.71073). Data have been corrected for absorption using SADABS[10] area detector absorption correction program. Using Olex2, the structure was solved with the SHELXT structure solution

program using Direct Methods and refined with the SHELXL refinement package using least squares minimization. All non-hydrogen atoms were refined with anisotropic thermal parameters. Initial solution and refinement of the structure were poor. The structure was treated as a two-component pseudo-merohedral twin. Using the TwinRotMat package contained within the PLATON program the suggested twin law of [0 -1 0 -1 0 0 0 0 -1] was applied. Subsequent model refinement led to a BASF of 0.4683(5) and resulted in a significant reduction in the R_1 statistic. After application of the twin law the hydrogen atoms of the investigated structure were located from difference Fourier maps. Ultimately their positions were placed in geometrically calculated positions and refined using a riding model. Isotropic thermal parameters of the placed hydrogen atoms were fixed to 1.2 times the *U* value of the atoms they are linked to (1.5 times for methyl groups). Hydrogen atoms connected to heteroatoms were located from the difference map, placed, and refined. Calculations and refinement of structures were carried out using APEX4, SHELXTL, and Olex2 software.

Experimental:

Synthesis of Fe(^{PPh}PDI)Cl$_2$ (1). In a Schlenk flask under inert atmosphere (N_2), [(ArN═C(CH_3))C_2H_3N((CH_3)C═O] (0.75 g, 2.3 mmol) and one equivalent $FeCl_2$ (0.32 g, 2.5 mmol) were dissolved in anhydrous EtOH. One equivalent of 2-(diphenylphosphino)ethylamine (0.59 g, 2.5 mmol) was dissolved in EtOH in a vial, which was fitted with a septum. The Schlenk flask was brought out of the glovebox and placed under a nitrogen atmosphere and heated to 40 °C while stirring. The 2-(diphenylphosphino)ethylamine was added to the reaction *via* syringe. The resulting blue solution was heated to 60 °C and stirred overnight. The solvent was evaporated and the Schlenk flask was brought into the glovebox, where the blue solid was redissolved in CH_2Cl_2. This solution was filtered through celite, dried via vacuum and triturated with Et_2O. Solid was then dried via vacuum and redissolved in CH_2Cl_2 before being layered with Et_2O to produce X-ray quality crystals (**1**) (1.15 g, 75% yield). FT-IR: $\nu_{C=N}$ = 1584 cm^{-1}. ^{1}H NMR (CD_2Cl_2) δ: 155.34, 82.41, 39.24, 17.42, 7.26, 6.58, 5.12, 1.97, 0.48, -6.97, -10.16, -13.10, -27.52 ppm. Mössbauer: δ = 0.859(2) mm s^{-1}; Δ*E* = 0.979(4) mm s^{-1}. μ_{eff} = 5.78 μ_B Anal. Calcd for $C_{35}H_{40}Cl_2FeN_3P$: C, 63.65; H, 6.10; N, 6.36. Found: C, 63.13; H, 6.45; N, 5.84

Synthesis of Fe(^{PPh}PDI)(CO) (2). 0.19 g (0.29 mmol) **1** and 2.1 equivalents of samarium(II) iodide (SmI_2) were added to a side-arm Schlenk tube with a stir bar under inert atmosphere (N_2). The concentration of SmI_2 in THF (~0.1 M) was confirmed via UV-Vis. Upon adding the SmI_2, the solution immediately changed from blue to dark red/brown. The headspace of the tube was evacuated and brought out of the glovebox, where it was charged with one equivalent of carbon monoxide (CO) (7 mL@1 atm). The red solution was stirred vigorously overnight, after which it was dried and brought into the glovebox. The brown solid was redissolved in Et_2O and filtered through celite. The solution was dried, redissolved in Et_2O, and filtered through an alumina plug. Slow evaporation yielded X-ray quality crystals of **2** (0.124 g, 70% yield). Mössbauer: δ = 0.213(1) mm s^{-1}; Δ*E* = 0.704(2) mm s^{-1}. FT-IR: ν_{CO} = 1856 cm^{-1}. ^{1}H NMR (CD_2Cl_2) δ: 8.16 (dd, 2H), 7.41 (t, 1H), 7.34 (t, 1H), 7.20 (m, 3H), 7.13 (dd, 2H), 7.03 (t, 2H), 6.92 (t, 3H), 6.69 (t, 2H), 4.29 (m, 1H), 3.47 (m, 1H), 2.96 (m, 2H), 2.69 (s, 3H), 2.59 (td, 1H), 2.30 (s, 3H), 2.08 (quin, 1H), 1.04 (d, 3H), 0.83 (d, 3H), 0.64 (d, 3H), 0.09 (d, 3H). ^{13}C NMR (CD_2Cl_2) δ: 154.36, 152.99, 150.77, 147.04, 145.56, 141.70, 141.46, 136.63, 135.15, 134.87, 132.48, 129.18, 128.66, 128.35, 125.61, 124.34, 123.55, 119.30, 118.58, 114.61, 66.06, 50.33, 36.53, 36.29, 27.88, 27.13, 25.35, 25.03, 24.39, 22.49, 17.18, 15.29. ^{31}P NMR (CD_2Cl_2) δ: 64 ppm. Anal. Calcd for $C_{36}H_{40}FeN_3OP$: C, 70.02; H, 6.53; N, 6.80. Found: C, 69.82; H, 6.36; N, 6.40.

Synthesis of (2) with NaHg. 0.50 g (0.76 mmol) **1** and 2.1 equivalents of sodium amalgam (NaHg) (5% Na) (0.93 g NaHg, 1.6 mmol Na) dissolved in CH_2Cl_2 in a Fisher Porter (FP) tube with a stir bar under inert atmosphere. The FP tube was fitted with a pressure gauge and brought out of the glovebox, where it was charged with 40 psi of carbon monoxide (CO). The green solution was stirred vigorously for 48 h, during which it turned red/brown. The solvent was evaporated via vacuum and brought into the glovebox (N_2). The brown solid was redissolved in Et_2O and filtered through celite. Separation of products was attempted by filtering through an alumina plug, but was unsuccessful. (Fig S9) FT-IR ν_{CO} = 1945, 1882, 1855 cm^{-1}.

Synthesis of 2 with CO$_2$. The procedure for the CO reduction reaction with 100 mg (0.151 mmol) Fe(^{PPh}PDI)Cl$_2$ (**1**) and two equivalents SmI_2 was followed substituting CO_2 in place of CO. The isolated yield was 20.4 mg, 0.033 mmol (22%) of crystalline Fe(^{PPh}PDI)(CO) (**2**).

CO_2 Reduction with Four Equivalents SmI_2. 100 mg (0.151 mmol) Fe(^{PPh}PDI)Cl_2 (**1**) was dissolved in 10 mL THF in a Schlenk tube producing a blue solution. One freeze pump thaw cycle was performed. While the solution was frozen and under static vacuum, 20 mL CO_2 (0.893 mol) was added to the tube via syringe. The solution was allowed to thaw and mix with no observable color change. The tube was then placed in an LN_2 bath to freeze the solution and condense any CO_2 from the headspace. While frozen, 6.8 mL of a 0.9 M SmI_2 (6.12 mmol) in THF was added slowly via syringe. The solution was then allowed to thaw to room temp where it turned green and then red over 20 minutes. The solution was allowed to stir overnight. The THF was removed via vacuum and the tube was brought into the glovebox where the resultant red/brown solid was redissolved in Et_2O, filtered through a neutral alumina plug, and allowed to slowly evaporate resulting in a 71% yield (66 mg, 0.107 mmol) of crystalline Fe(^{PPh}PDI)(CO) (**2**).

Synthesis of [Fe(^{PPh}PDI)(NO)][X] (X = BPh_4^- or PF_6^-) (**3**). The procedure was performed using either [$HNEt_3$][BPh_4] or [$HNEt_3$][PF_6]. For simplicity only the synthesis utilizing [$HNEt_3$][BPh_4] is reported here. 0.0771 g (0.12 mmol) **2** and two equivalents [$HNEt_3$][BPh_4] (0.1065 g, 0.25 mmol) were dissolved in 7 mL THF in a side arm flask and stirred. One equivalent sodium nitrite ($NaNO_2$) (0.0088 g, 0.13 mmol) was dissolved in a minimal amount of MeOH and added dropwise to the reaction flask, which was then immediately sealed and allowed to stir at room temperature. After an hour, the color had changed from red to brownish green. After 24 hr, the headspace of the reaction was sampled for Gas FT-IR analysis before drying the solution *in vacuo*. The red/black solid was triturated in Et_2O overnight to remove unreacted (**2**). The solid was then dissolved in CH_2Cl_2 and the green solution was filtered through celite, leaving a white precipitate ($NaBPh_4$). The CH_2Cl_2 solution was layered with either pentane or Et_2O to produce crystals of [Fe(^{PPh}PDI)(NO)][BPh_4] (**3**) (0.739 g, 98% yield). Mössbauer: δ = 0.127(2) mm s^{-1}; ΔE = 1.012(6) mm s^{-1}. FT-IR ν_{14NO} = 1707 cm^{-1}. ^{1}H NMR (CD_2Cl_2) δ: 8.05 (dd, 2H), 7.82 (t, 1H), 7.45 (t, 2H), 7.35 (s, 9H), 7.31 (m, 2H), 7.21 (t, 2H), 7.10 (d, 1H), 7.01 (m, 11H), 6.85 (m, 6H), 4.13 (m, 1H), 3.53 (m, 1H), 2.68 (m, 1H), 2.64 (s, 3H), 2.50 (td, 1H), 2.43 (s, 3H), 2.22 (sept, 2H), 1.07 (d, 3H), 0.74 (t, 6H), 0.14 (d, 3H). ^{13}C NMR (CD_2Cl_2) δ: 171.92, 165.04, 164.65, 163.87, 151.54, 150.69, 147.31, 140.83, 139.82, 136.36, 133.43, 132.25, 131.70, 131.32, 129.87, 129.54, 128.76, 128.37, 127.90, 126.43, 126.00, 125.40, 125.20, 124.79, 122.14, 51.34, 32.92, 28.76, 27.45, 25.21, 24.50, 22.17, 18.41, 16.68 ppm. ^{31}P NMR (CD_2Cl_2) δ: 50 ppm. Anal. Calcd for $C_{59}H_{60}BFeN_4OP$: C, 75.49; H, 6.44 N, 5.97. Found: 75.85; H, 6.59; N, 5.24.

Synthesis of ^{15}N Isotope [Fe(^{PPh}PDI)(NO)][BPh_4]. The procedure above was repeated with $Na^{15}NO_2$ in place of $NaNO_2$. The CH_2Cl_2 solution was layered with either Et_2O to produce X-ray quality crystals of [Fe(^{PPh}PDI)(^{15}NO)][BPh_4] (**3**) FT-IR ν_{15NO} = 1675 cm^{-1}. ^{1}H NMR (CD_2Cl_2) δ: 8.05 (dd, 2H), 7.82 (t, 1H), 7.45 (t, 2H), 7.35 (s, 9H), 7.31 (m, 2H), 7.21 (t, 2H), 7.10 (d, 1H), 7.01 (m, 11H), 6.85 (m, 6H), 4.13 (m, 1H), 3.53 (m, 1H), 2.68 (m, 1H), 2.64 (s, 3H), 2.50 (td, 1H), 2.43 (s, 3H), 2.22 (sept, 2H), 1.07 (d, 3H), 0.74 (t, 6H), 0.14 (d, 3H). ^{31}P NMR (CD_2Cl_2) δ: 50 ppm.

Attempted Synthesis of [Fe(^{PPh}PDI)$(NO)_2$][BPh_4]. The synthesis of [Fe(^{PPh}PDI)$(NO)_2$][BPh_4] was performed via two different procedures. The first attempt used 0.1027 g (0.17 mmol) **2** and four equivalents [$HNEt_3$][BPh_4] (0.2818 g, 0.67 mmol) which were dissolved in 6 mL THF. While stirring, two equivalents $NaNO_2$ (0.0261 g, 0.38 mmol) in 1 mL MeOH were added. The solution was stirred overnight, changing from red/brown to green. The solid was characterized as (**3**) through FT-IR ν_{14NO} = 1708 cm^{-1}. ^{1}H NMR (500MHz, CD_2Cl_2) δ: 8.05 (dd, 2H), 7.82 (t, 1H), 7.45 (t, 2H), 7.35 (s, 9H), 7.31 (m, 2H), 7.21 (t, 2H), 7.10 (d, 1H), 7.01 (m, 11H), 6.85 (m, 6H), 4.13 (m, 1H), 3.53 (m, 1H), 2.68 (m, 1H), 2.64 (s, 3H), 2.50 (td, 1H), 2.43 (s, 3H), 2.22 (sept, 2H), 1.07 (d, 3H), 0.74 (t, 6H), 0.14 (d, 3H). ^{31}P NMR (CD_2Cl_2) δ: 50 ppm.

The second procedure utilized (**3**), (0.1151 g, 0.12 mmol) and two equivalents $[HNEt_3][BPh_4]$ (0.1150 g, 0.27 mmol) which were dissolved in 6 mL THF. While stirring, one equivalent $NaNO_2$ (0.0180 g, 0.26 mmol) in 1 mL MeOH, was added. After stirring for two days, no color change was observed and no change in the IR was observed. After heating to 80 ^{o}C for 16 h, some decomposition was observed. The solid was characterized as starting (**3**) through FT-IR ν_{14NO} = 1707 cm^{-1}. ^{1}H NMR (CD_2Cl_2) δ: 8.05 (dd, 2H), 7.82 (t, 1H), 7.45 (t, 2H), 7.35 (s, 9H), 7.31 (m, 2H), 7.21 (t, 2H), 7.10 (d, 1H), 7.01 (m, 11H), 6.85 (m, 6H), 4.13 (m, 1H), 3.53 (m, 1H), 2.68 (m, 1H), 2.64 (s, 3H), 2.50 (td, 1H), 2.43 (s, 3H), 2.22 (sept, 2H), 1.07 (d, 3H), 0.74 (t, 6H), 0.14 (d, 3H). ^{31}P NMR (CD_2Cl_2) δ: 50 ppm.

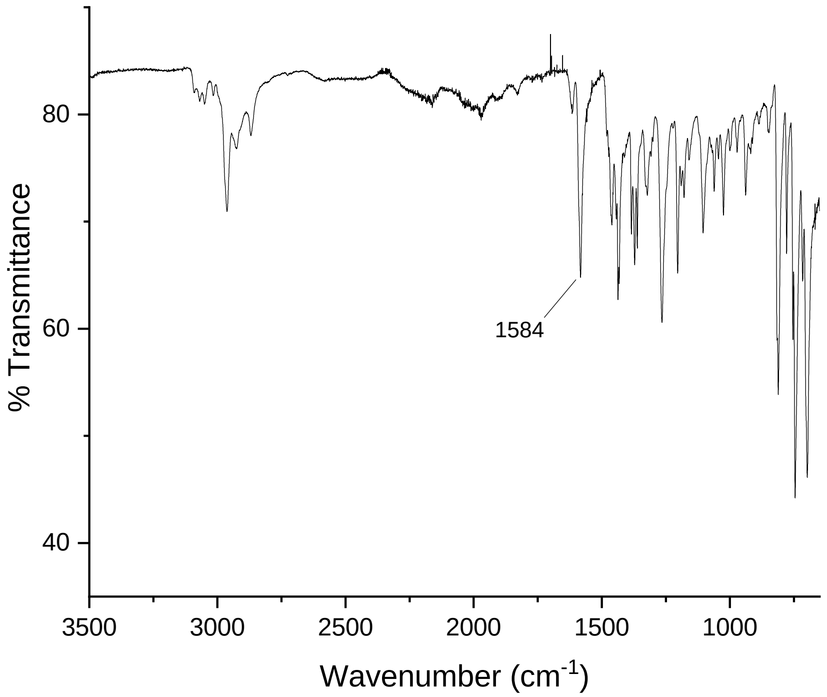

Figure S1. FTIR Spectrum of Fe(^{PPh}PDI)Cl_2 (**1**).

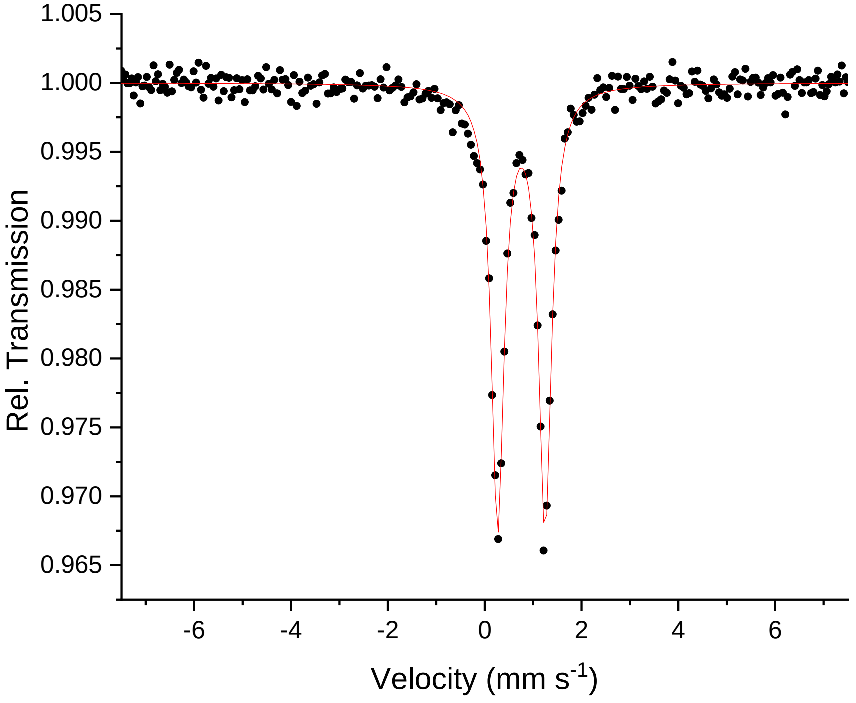

Figure S2. Mössbauer of Fe(^{PPh}PDI)Cl_2 (**1**). δ = 0.859(2) mm s^{-1}; ΔE = 0.979(4) mm s^{-1}

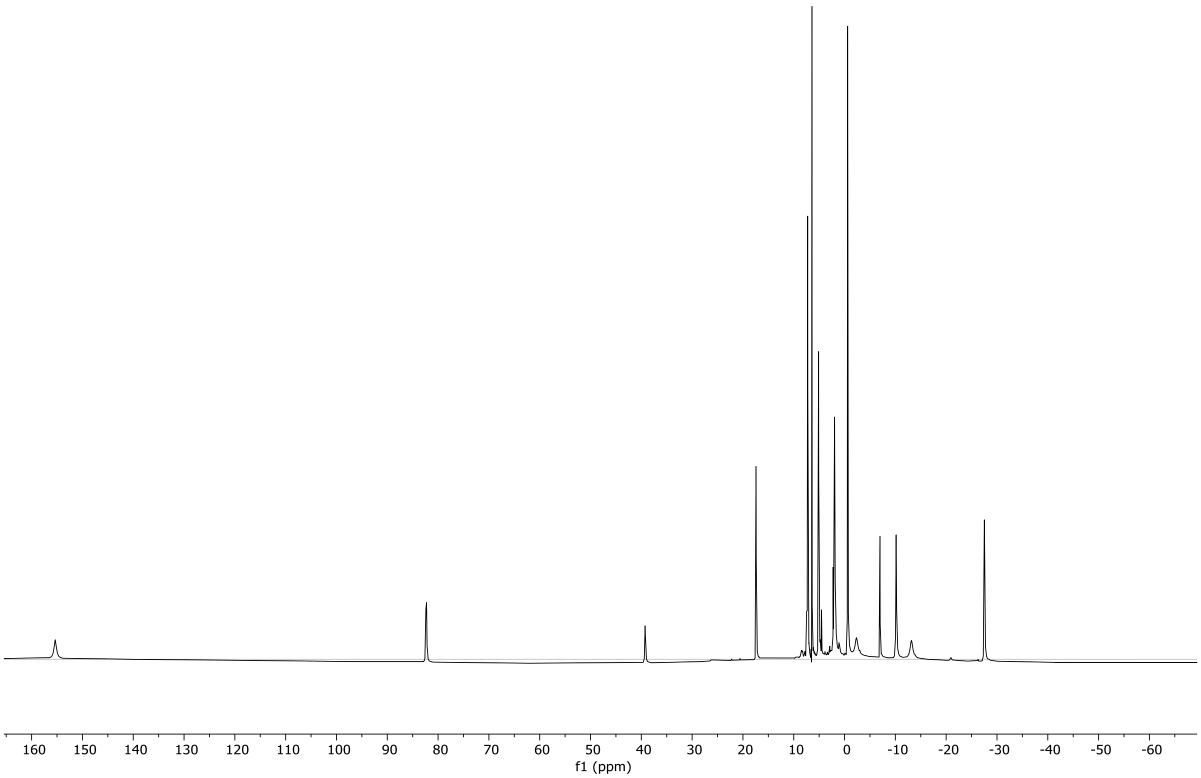

Figure S3. ^{1}H NMR Spectrum of Fe(^{PPh}PDI)Cl_2 (**1**).

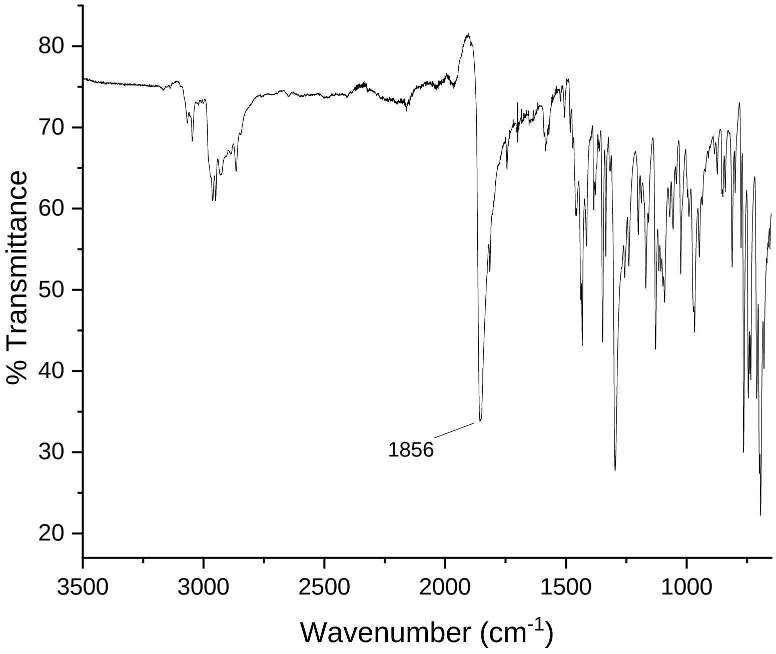

Figure S4. FTIR Spectrum of Fe(^{PPh}PDI)(CO) (**2**).

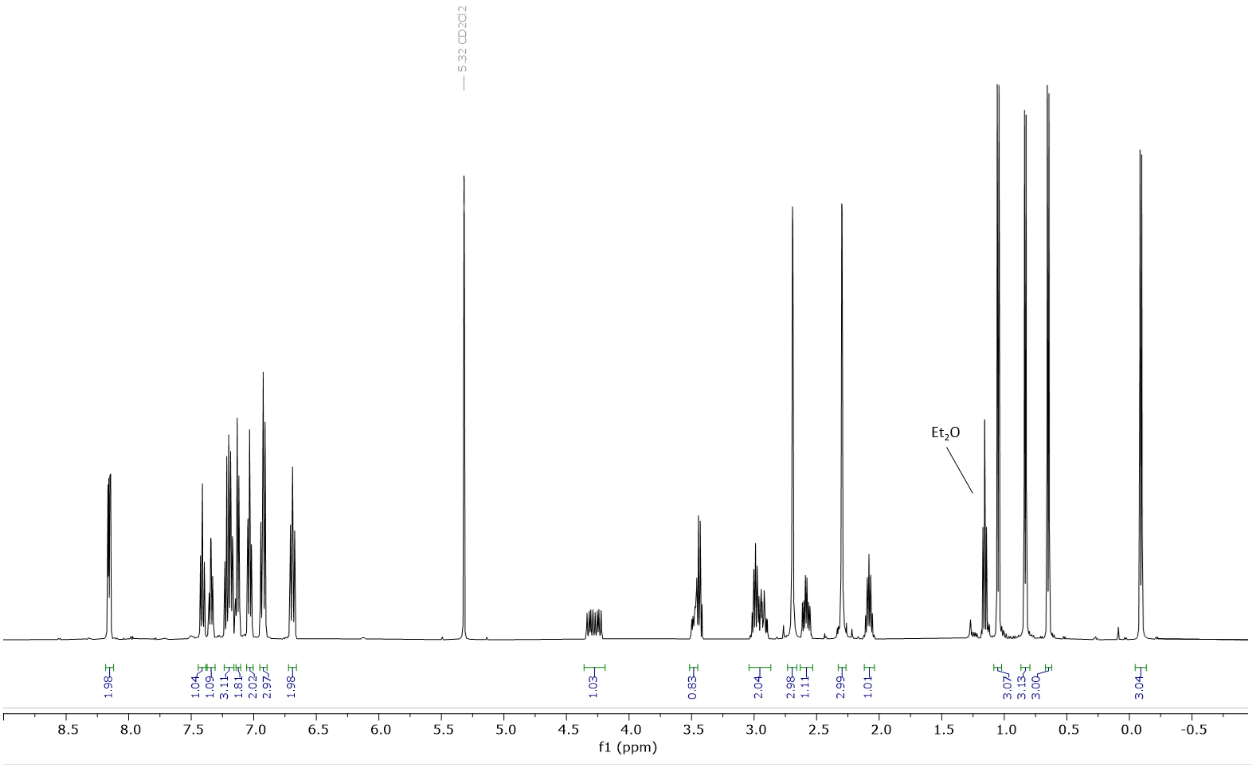

Figure S5. ^{1}H NMR Spectrum of Fe(^{PPh}PDI)(CO) (**2**).

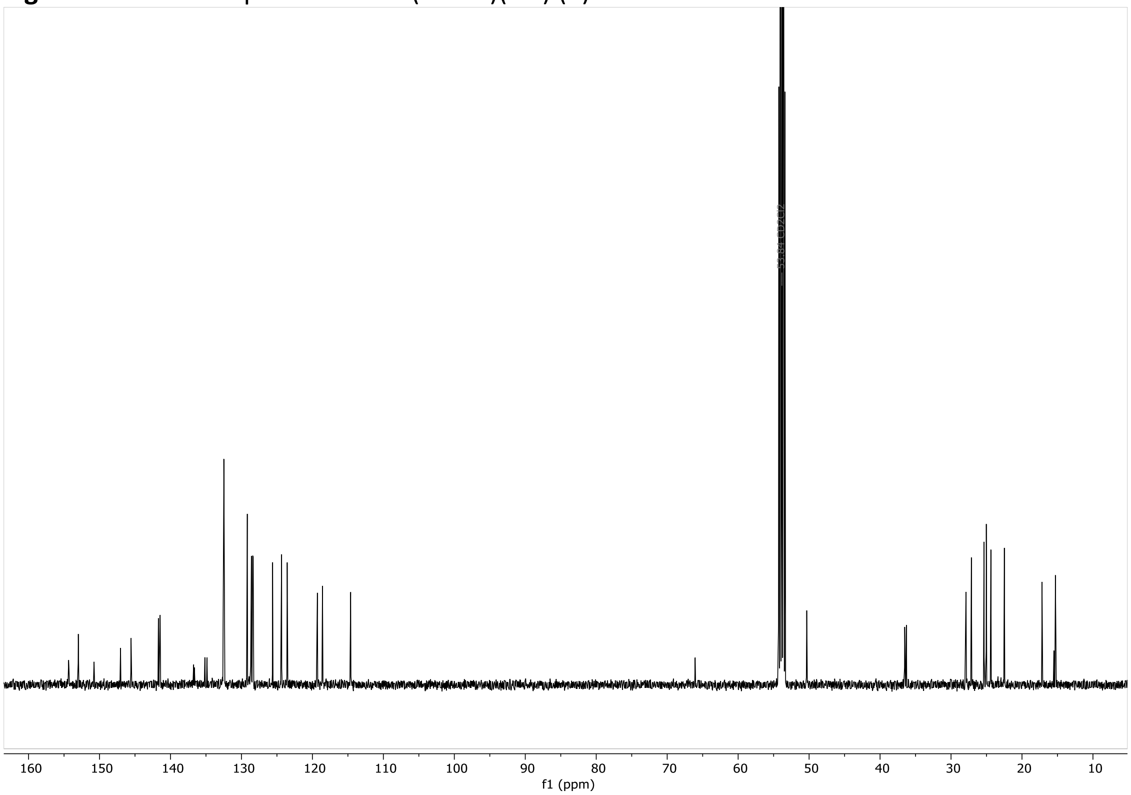

Figure S6. ^{13}C NMR Spectrum of Fe(^{PPh}PDI)(CO) (**2**).

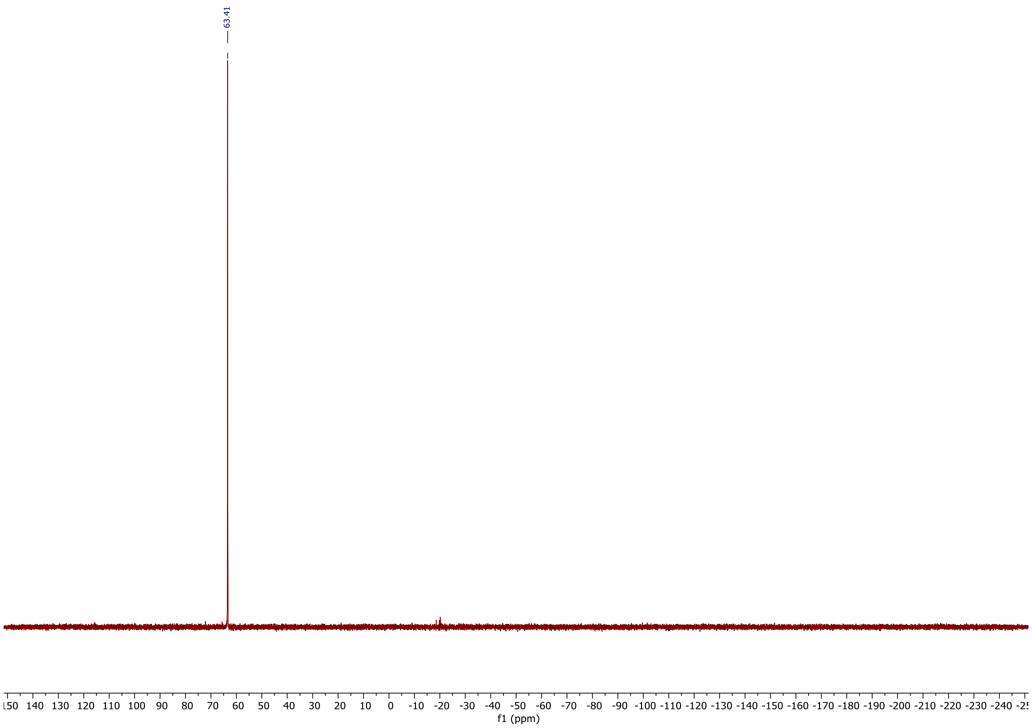

Figure S7. ^{31}P NMR Spectrum of Fe(^{PPh}PDI)(CO) (**2**).

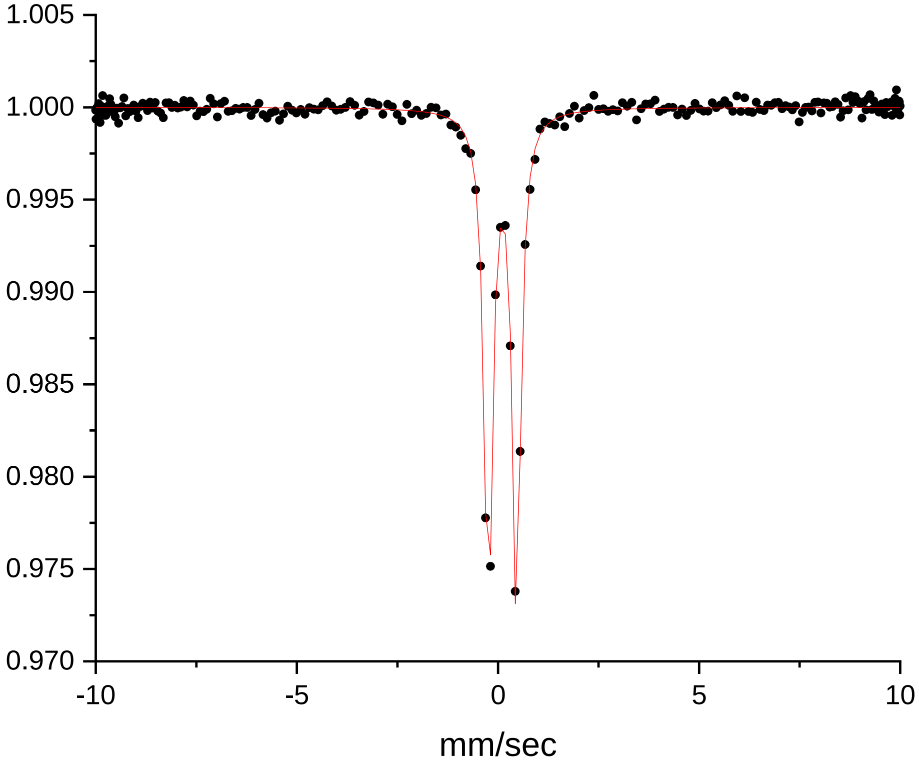

Figure S8. Mössbauer of Fe(^{PPh}PDI)(CO) (**2**). δ = 0.213(1) mm s^{-1}; Δ*E* = 0.704(2) mm s^{-1}.

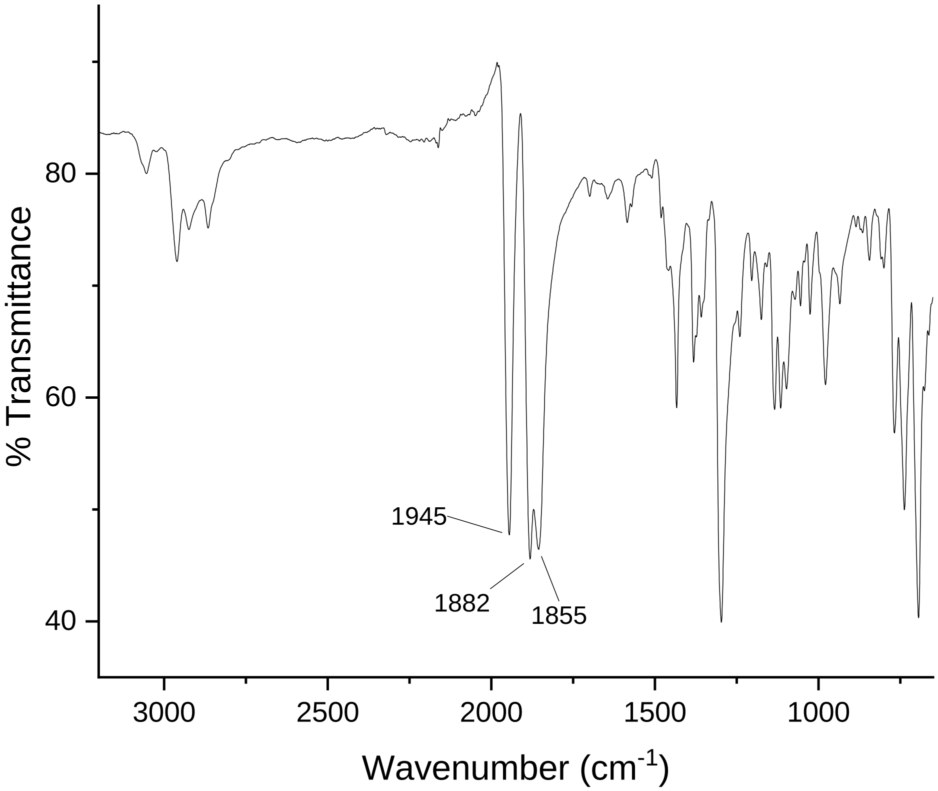

Figure S9. FT-IR Spectrum of reaction mixture of Fe(^{PPh}PDI)Cl_2 (**1**) and NaHg under CO atmosphere. The spectrum reveals a mixture of Fe(^{PPh}PDI)(CO) (**2**) and Fe(^{PPh}PDI)$(CO)_2$.

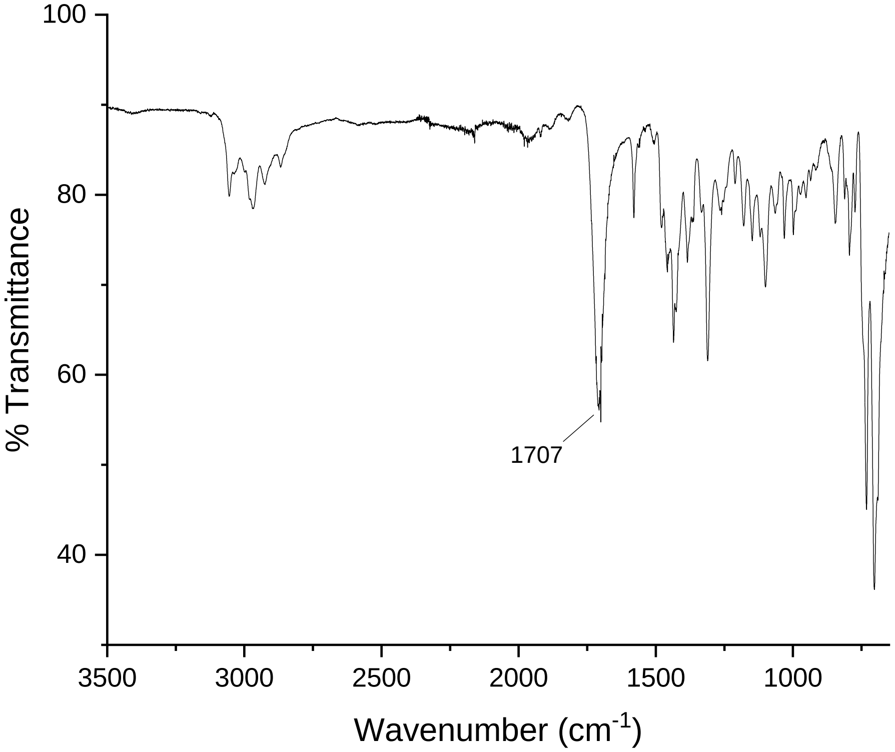

Figure S10. FTIR Spectrum of [Fe(^{PPh}PDI)(NO)][BPh_4] (**3**).

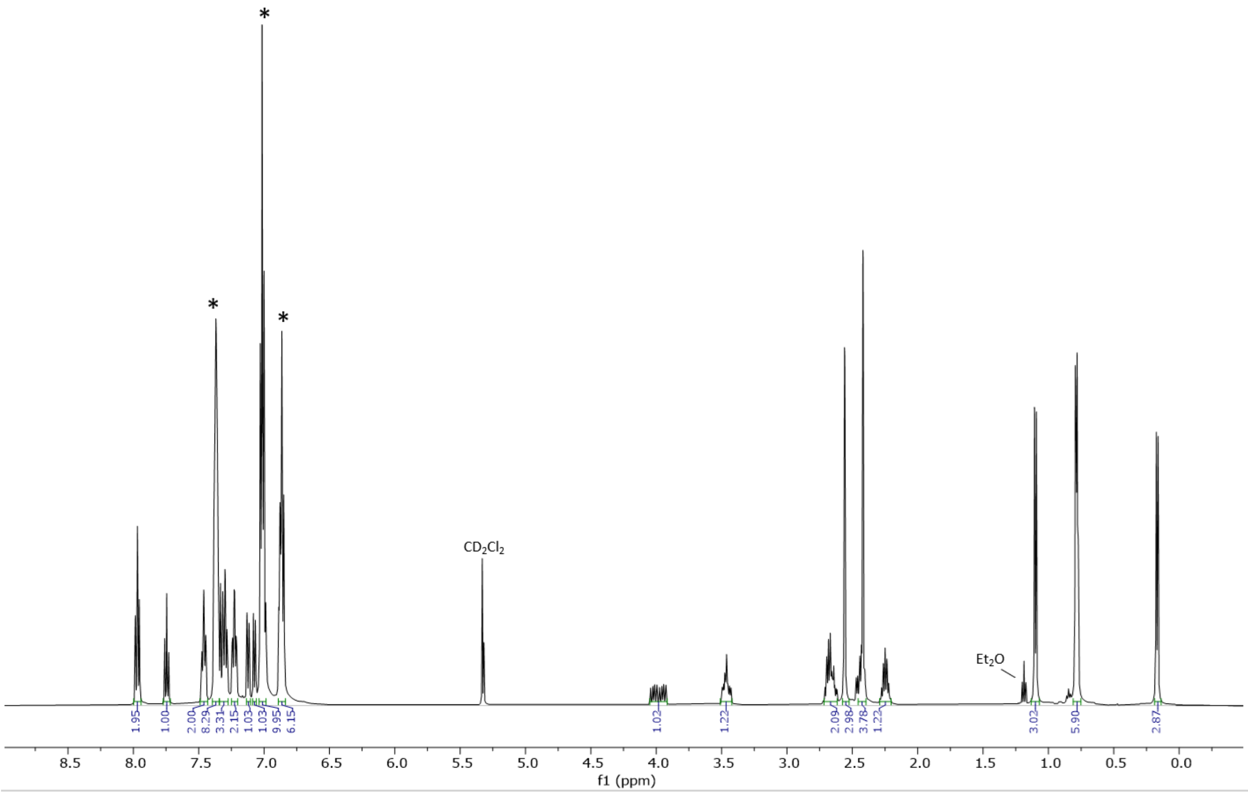

Figure S11. ^{1}H NMR Spectrum of [Fe(^{PPh}PDI)(NO)][BPh$_4$] (**3**). * BPh$_4$ anion.

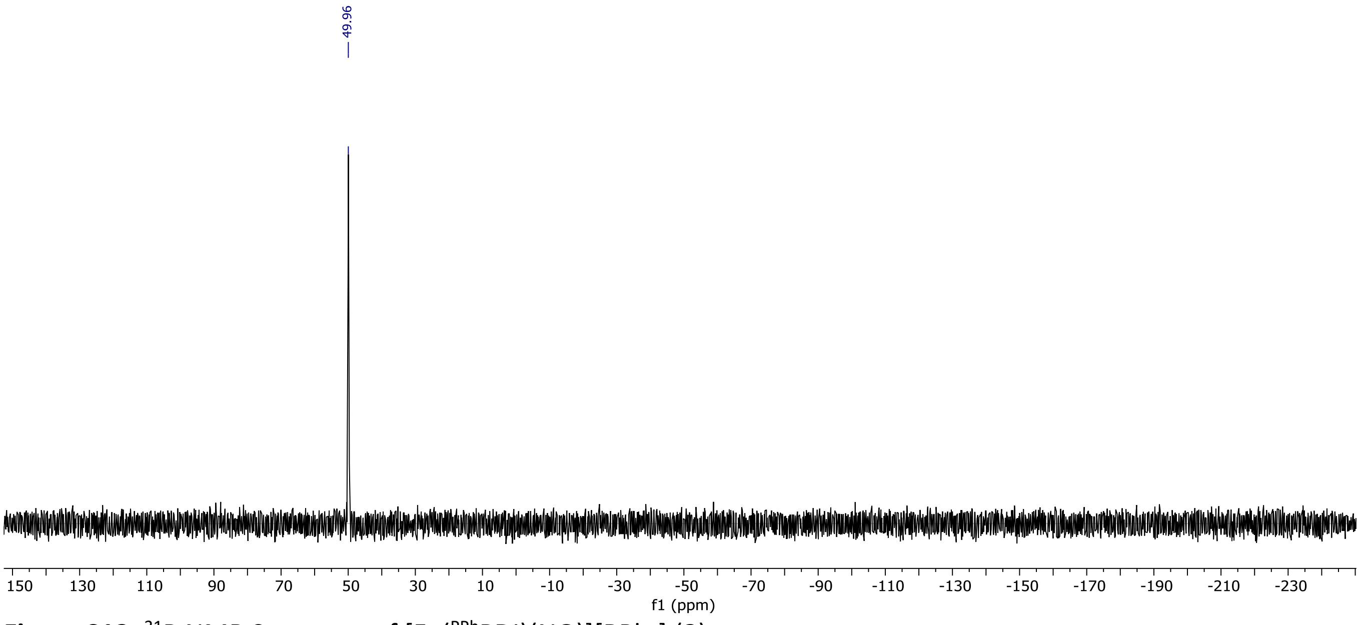

Figure S12. ^{31}P NMR Spectrum of [Fe(^{PPh}PDI)(NO)][BPh$_4$] (**3**).

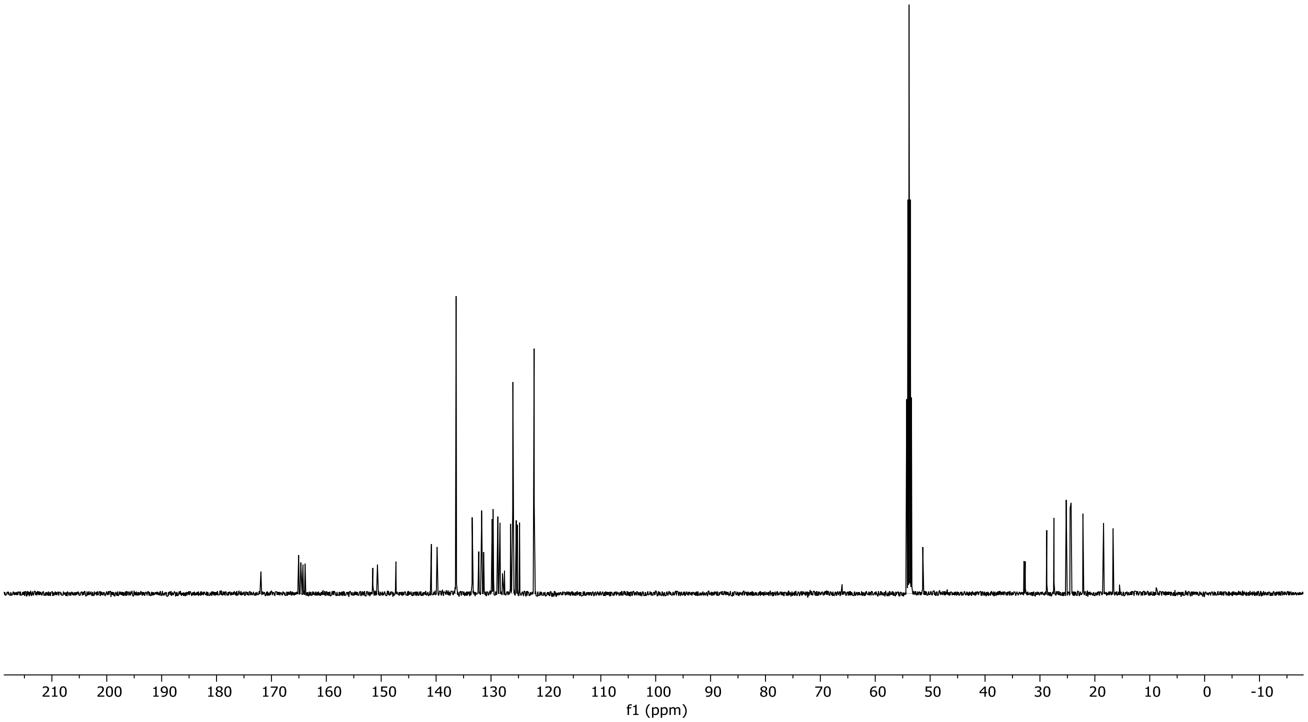

Figure S13. ^{13}C NMR Spectrum of $[Fe(^{PPh}PDI)(NO)][BPh_4]$ (**3**).

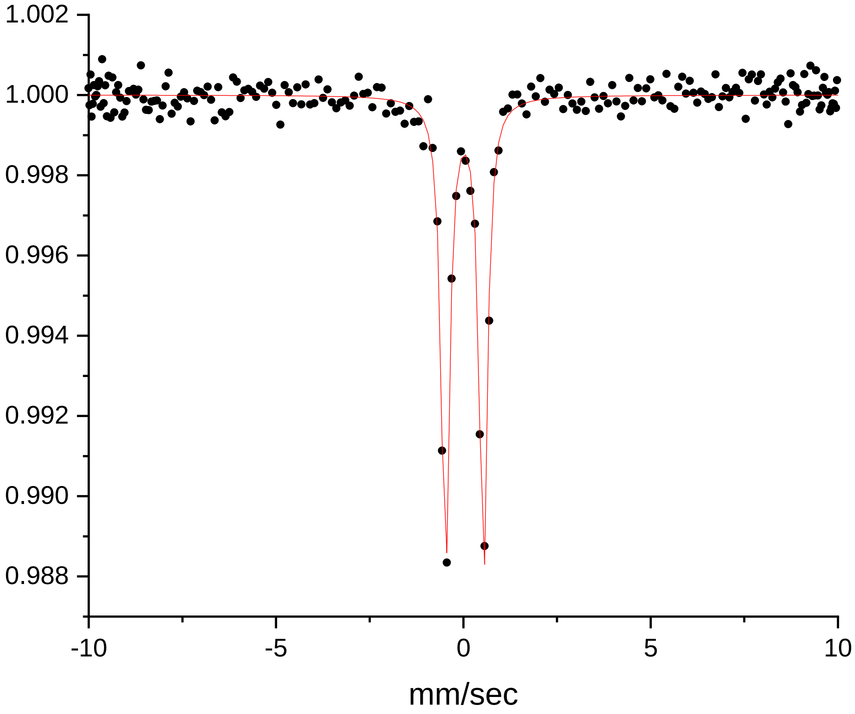

Figure S14. Mössbauer of $[Fe(^{PPh}PDI)(NO)][BPh_4]$ (**3**). $\delta = 0.127(2)$ mm s^{-1}; $\Delta E = 1.012(6)$ mm s^{-1}

Table S1. Geometric parameters in the BS(2,2)-PBE0/def2-TZVP(-f) optimized geometries MNIC structure.

	**3**	**4**	**1a**
Bond Length	Length (Å)	Length (Å)	Length (Å)
Fe—NO	1.7124	1.7622	1.7472
N—O	1.1580	1.1645	1.1672
Fe—$N_{pyridyl}$	1.8566	1.8413	1.8387
Fe—N_{amine}	1.9429, 2.0467	1.9372, 2.0706	1.8989, 2.0595
Fe—Pnictogen	2.3635	2.0710	2.1014
Bond Angle	Angle (°)	Angle (°)	Angle (°)
Fe-N-O	162.2	148.2	147.5

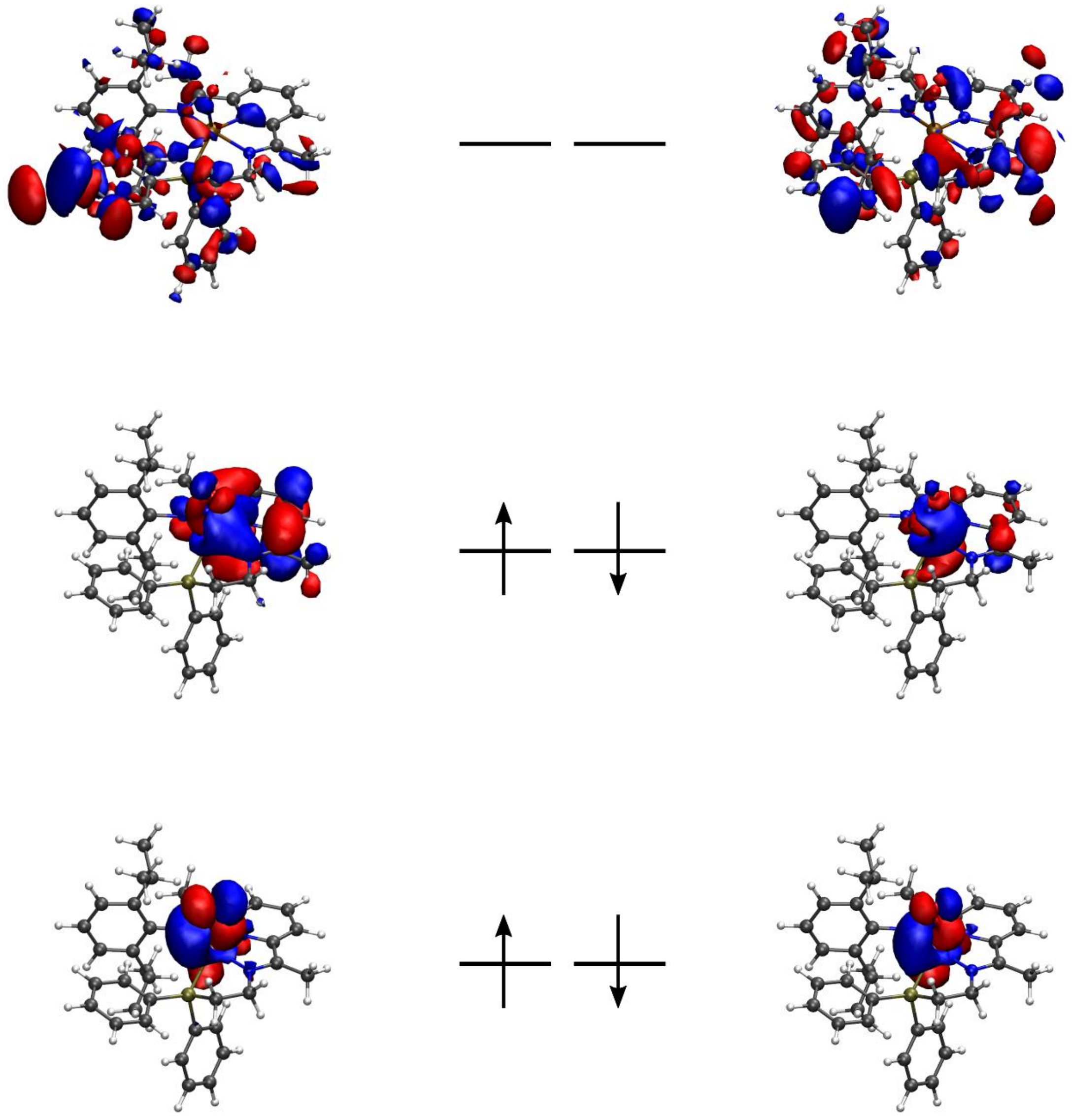

Figure S15. BS(2,2) frontier corresponding orbitals of **3** at the PBE0/def2-TZVP(-f) level of theory

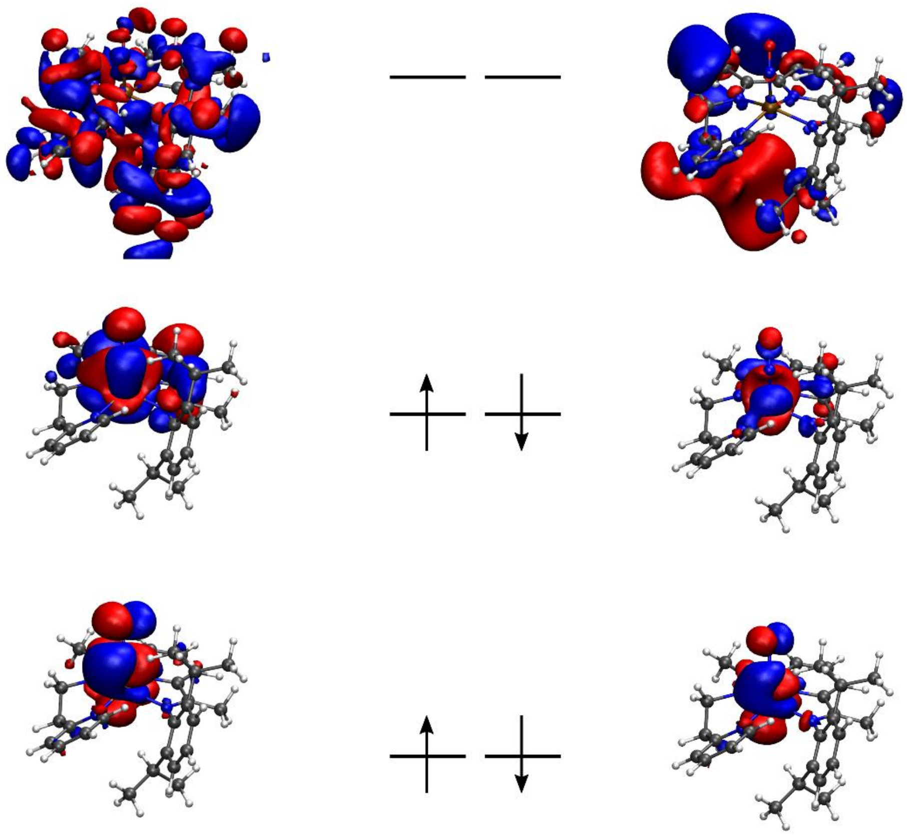

Figure S16. BS(2,2) frontier corresponding orbitals of **4** at the PBE0/def2-TZVP(-f) level of theory

Optimized geometries of **3**, **4**, and **1a** at the BS-PBE0/def2-TZVP(-f) level.

```
82
BS(2,2)-PBE0/def2-TZVP(-f) optimized geometry of 3
  Fe      8.181926      3.887782      5.166465
  P       9.702823      4.726267      6.769541
  N       7.227723      4.435394      3.670847
  N       9.225411      2.964861      3.667047
  O       6.730788      1.956751      6.654981
  N       7.472247      2.591802      6.032101
  C       6.202482      5.316063      3.833797
  C      10.399488      2.154964      3.820589
  C       7.473133      3.868987      2.465169
  C      10.274332      0.807737      4.195275
  C      12.785695      1.944010      3.753481
  H      13.762992      2.374326      3.572030
  C      11.828174      4.191360      3.199151
  H      10.867020      4.693986      3.333666
  C       8.941753      0.116805      4.393055
  H       8.150290      0.852953      4.238952
  C      11.656855      2.742126      3.604738
  C      10.433062      6.340915      6.323474
  C      12.686691      0.618595      4.133751
  H      13.580369      0.016515      4.248073
  C       5.364265      5.615952      2.762702
  H       4.543753      6.310501      2.887441
  C       8.638027      3.011753      2.505079
  C      11.439940      0.065281      4.359275
  H      11.362744     -0.972564      4.662337
  C      10.924785      3.814056      7.755173
  C       9.059416      2.270723      1.284057
  H      10.008983      1.761125      1.426382
  H       9.144506      2.953970      0.436537
  H       8.300472      1.527542      1.023697
  C       9.958265      6.980146      5.179265
  H       9.208517      6.492850      4.565294
  C       6.169736      5.823483      5.167751
  C      11.583942      2.720905      7.199142
  H      11.375221      2.418379      6.181635
  C      11.415327      6.966167      7.089513
  H      11.817974      6.476385      7.967591
  C      12.850483      4.906723      4.076651
  H      12.632690      4.780885      5.138296
  H      12.852089      5.976455      3.856556
  H      13.860442      4.532404      3.894236
  C       6.661758      4.136358      1.383161
  H       6.858159      3.672712      0.425429
  C      11.416691      8.843533      5.584973
  H      11.805952      9.811386      5.292114
  C      11.903400      8.210203      6.720587
  H      12.667885      8.686874      7.322576
```

C	5.589573	5.018155	1.536361
H	4.946188	5.241964	0.695367
C	8.797409	-0.440496	5.808888
H	9.486710	-1.270583	5.977322
H	7.784766	-0.816652	5.971455
H	9.002754	0.318312	6.565073
C	8.749507	-0.988162	3.355346
H	8.870793	-0.607454	2.338828
H	7.752420	-1.427029	3.441832
H	9.475590	-1.792240	3.494742
C	12.227432	4.307369	1.727457
H	13.164588	3.777682	1.538774
H	12.377867	5.353962	1.451708
H	11.471878	3.885265	1.062077
C	10.442937	8.226436	4.814681
H	10.062167	8.712569	3.924031
C	11.202557	4.176470	9.075958
H	10.700006	5.018484	9.536677
C	5.178423	6.820173	5.652499
C	12.123428	3.459960	9.821427
H	12.339617	3.759515	10.840223
C	12.501226	2.001474	7.951292
H	13.002627	1.150244	7.507504
C	12.773517	2.369400	9.258738
H	13.487484	1.803660	9.845506
C	8.408101	5.137237	8.008838
C	7.237524	5.796260	7.306721
N	7.119018	5.323195	5.930892
H	4.555044	7.194099	4.843086
H	5.674644	7.676488	6.115932
H	4.522618	6.374730	6.407208
H	6.310416	5.591469	7.852632
H	7.374168	6.883038	7.297792
H	8.788147	5.781667	8.801702
H	8.112610	4.186742	8.458997

69
BS(2,2)-PBE0/def2-TZVP(-f) optimized geometry of **4**

Fe	0.014071	-0.056932	-0.071561
N	2.084559	-0.045322	-0.049211
C	2.604942	1.148092	-0.089215
C	1.636085	2.209314	-0.249274
N	0.372812	1.729067	-0.339488
C	-0.678088	2.566360	-0.564221
C	-1.875262	1.828964	-0.828614
N	-1.669788	0.529965	-0.828429
C	-2.696307	-0.381951	-1.278067
C	-2.075440	-1.432066	-2.190920
C	-1.275318	-2.450422	-1.440971
N	-0.359673	-2.038476	-0.543797
C	0.281583	-2.965604	0.180244

H	0.995658	-2.597008	0.903868
C	0.065534	-4.321778	0.042501
H	0.610439	-5.016065	0.667744
C	-0.851195	-4.751473	-0.901229
C	-1.525250	-3.800996	-1.643704
H	-2.265014	-4.095877	-2.376794
H	-1.053110	-5.805848	-1.050695
H	-2.866477	-1.947436	-2.737652
H	-1.449019	-0.926641	-2.933905
H	-3.472740	0.151645	-1.828951
H	-3.173187	-0.864847	-0.417853
C	-3.187581	2.478106	-1.112189
H	-3.416696	2.476173	-2.182561
H	-3.182961	3.513880	-0.776802
H	-4.000767	1.966983	-0.596323
C	-0.476518	3.942070	-0.591221
H	-1.313699	4.611488	-0.740146
C	0.806054	4.445874	-0.458388
C	1.880318	3.566427	-0.312899
H	2.892745	3.942612	-0.245039
H	0.978416	5.513965	-0.492842
C	4.057671	1.464092	-0.001769
H	4.272141	2.033156	0.906476
H	4.355133	2.086388	-0.849278
H	4.664061	0.562329	0.003987
C	2.931626	-1.172296	0.187924
C	3.228953	-2.020050	-0.892593
C	2.768074	-1.684314	-2.294935
H	1.898936	-1.025947	-2.201734
C	2.336045	-2.902424	-3.100862
H	3.182865	-3.543664	-3.355972
H	1.881272	-2.581395	-4.041264
H	1.609016	-3.508788	-2.556543
C	3.853926	-0.906115	-3.036841
H	4.181129	-0.030102	-2.473839
H	3.489367	-0.565872	-4.009281
H	4.729874	-1.538312	-3.201359
C	4.017994	-3.137874	-0.652182
C	4.475183	-3.429337	0.621893
H	5.083989	-4.308830	0.793433
C	4.165143	-2.587736	1.673344
C	3.401597	-1.437823	1.486490
C	3.127269	-0.542618	2.679437
H	2.566052	0.329621	2.337194
C	2.275336	-1.252680	3.731256
H	1.334980	-1.621728	3.317043
H	2.036466	-0.567713	4.547290
H	2.812502	-2.102880	4.158507
C	4.423812	-0.043212	3.321713
H	4.979703	-0.863568	3.780949
H	4.197880	0.677995	4.110365
H	5.083844	0.440864	2.599167

H	4.537159	-2.820303	2.664722
H	4.283225	-3.791884	-1.474052
N	-0.443458	-0.097154	1.629738
O	-0.902581	0.457764	2.544831

55

BS(2,2)-PBE0/def2-TZVP(-f) optimized geometry of **1a**

Fe	9.007879	5.018283	10.261694
N	7.183875	5.852645	10.729084
N	8.029704	4.592401	8.764209
N	10.024951	3.554102	9.607768
C	5.155697	6.768173	9.634128
H	4.332387	6.206146	9.190985
H	4.858110	7.121034	10.618872
H	5.338316	7.638941	8.996345
C	6.392754	5.944571	9.698909
C	6.826378	5.172103	8.550252
C	6.143848	4.942043	7.373848
H	5.182017	5.404312	7.196608
C	6.701174	4.084552	6.422192
H	6.169715	3.880906	5.501755
C	7.918617	3.473414	6.667793
H	8.343865	2.791040	5.943492
C	8.579181	3.727070	7.865772
C	9.779313	3.143437	8.384076
C	10.664561	2.218337	7.628637
H	10.158605	1.796487	6.762421
H	11.548136	2.756069	7.268202
H	11.013718	1.392954	8.251429
O	10.253598	7.011408	8.736116
N	9.874669	6.412445	9.663463
N	10.044014	4.585932	12.038075
C	11.117936	3.067019	10.417467
H	12.039401	2.930403	9.843793
H	10.848144	2.087877	10.829883
C	11.343451	4.085690	11.519279
H	11.958953	3.663413	12.319359
H	11.869802	4.947489	11.105466
C	9.401391	3.567994	12.908336
H	9.418106	2.589253	12.429275
H	8.360794	3.865112	13.036424
C	10.140862	3.626764	14.244119
H	10.980917	2.930293	14.263171
H	9.480747	3.343639	15.064354
C	10.627429	5.085156	14.341728
H	10.131451	5.626976	15.145742
H	11.699250	5.134886	14.538238
C	10.270778	5.707222	12.983174
H	9.339471	6.269944	13.051464
H	11.040592	6.368341	12.584204
C	6.830041	6.464725	11.973552

C	6.130873	5.676508	12.907544
C	5.840715	6.243077	14.143364
H	5.297622	5.664565	14.880502
C	6.223388	7.538052	14.448585
H	5.979132	7.960251	15.416364
C	6.899295	8.296077	13.512167
H	7.180769	9.313716	13.756645
C	7.212343	7.785649	12.254053
C	5.639603	4.279791	12.569564
H	6.304179	3.870523	11.800579
C	4.224299	4.327006	11.987427

Table S2. Crystal data and structure refinement for **1** - **3**

**Compound**	**1**	**2**	**3[PF6]**
**Empirical formula**	$C_{35}H_{40}Cl_2FeN_3P$	$C_{36}H_{40}FeN_3OP$	$C_{35}H_{40}F_6FeN_4OP_2$
**Formula weight**	660.42	617.53	764.5
**Temperature/K**	100.0(2)	99.99(10)	100
**Crystal system**	monoclinic	monoclinic	triclinic
**Space group**	$P2_1/c$	$P2_1/c$	P-1
**a/Å**	22.1183(5)	17.1866(2)	13.1726(12)
**b/Å**	9.5982(2)	10.36160(10)	13.1878(11)
**c/Å**	15.8117(4)	18.5810(3)	21.6928(18)
**α/°**	90	90	77.828(4)
**β/°**	99.345(2)	108.086(2)	77.728(4)
**γ/°**	90	90	71.830(4)
**Volume/Å^3**	3312.21(13)	3145.43(8)	3456.0(5)
**Z**	4	4	4
**ρ_{calc}g/cm^3**	1.324	1.304	1.469
**μ/mm^{-1}**	0.693	0.563	0.597
**F(000)**	1384	1304	1584
**Crystal size/mm^3**	0.428 × 0.189 × 0.114	0.334 × 0.275 × 0.161	0.31 × 0.23 × 0.2
**Radiation**	MoKα (λ = 0.71073)	MoKα (λ = 0.71073)	MoKα (λ = 0.71073)
**2Θ range for data collection/°**	5.652 to 61.016	4.986 to 56.846	5.474 to 59.88
**Index ranges**	-31 ≤ h ≤ 31, -13 ≤ k ≤ 13, -22 ≤ l ≤ 21	-22 ≤ h ≤ 22, -13 ≤ k ≤ 13, -24 ≤ l ≤ 24	-18 ≤ h ≤ 18, -18 ≤ k ≤ 18, -30 ≤ l ≤ 30
**Reflections collected**	84820	163915	183454
**Independent reflections**	10063 [R_{int} = 0.0248, R_{sigma} = 0.0145]	7861 [R_{int} = 0.0246, R_{sigma} = 0.0073]	19708 [R_{int} = 0.0595, R_{sigma} = 0.0468]
**Data/restraints/parameters**	10063/0/385	7861/0/539	19708/0/899
**Goodness-of-fit on F^2**	1.074	1.027	1.103
**Final R indexes [I>=2σ (I)]**	R_1 = 0.0278, wR_2 = 0.0702	R_1 = 0.0258, wR_2 = 0.0688	R_1 = 0.0374, wR_2 = 0.0822
**Final R indexes [all data]**	R_1 = 0.0340, wR_2 = 0.0730	R_1 = 0.0276, wR_2 = 0.0700	R_1 = 0.0529, wR_2 = 0.0866
**Largest diff. peak/hole / e Å^{-3}**	0.64/-0.31	0.43/-0.30	0.47/-1.01

Table S3. NBO Charges and Widberg bond indices for **1a**, **3**, and **4**

**Complex**	**Hemilabile group**	**NBO charge**	**bond index**
**1a**	pyrrolidine	-0.25	0.2630
**4**	pyridine	-0.27	0.2745
**3**	phosphine	+0.52	0.4016

References:

[1] Bianchini, C.; Mantovani, G.; Meli, A.; Migliacci, F.; Zanobini, F.; Laschi, F.; Sommazzi, A. Oligomerisation of Ethylene to Linear α-Olefins by new Cs- and C1-Symmetric [2,6-Bis(imino)pyridyl]iron and -cobalt Dichloride Complexes. *Eur. J. Inorg. Chem.* **2003**, *2003*, 1620– 1631, DOI: 10.1002/ejic.200390213

[2] Bain, G. A.; Berry, J. F. Diamagnetic Corrections and Pascal's Constants. *J. Chem. Educ*. **2008**, *85*, 532−536 DOI: 10.1021/ed085p532

[3] CrysAlisPro, Rigaku Oxford Diffraction, version 171.41.85a, 2020

[4] CrysAlisPro, Rigaku Oxford Diffraction, version 171.40.55a, 2019

[5] SCALE3 ABSPACK – A Rigaku Oxford Diffraction program for Absorption Corrections, Rigaku Oxford Diffraction, 2017

[6] Sheldrick, G. M. SHELXT − Integrated space-group and crystal-structure determination. Acta Crystallogr., Sect. A: Found. Adv. 2015, A71, 3-8.

[7] Sheldrick, G. M. Crystal structure refinement with SHELXL. Acta Crystallogr., Sect. C: Struct. Chem. 2015, C71, 3-8.

[8] Dolomanov, O. V.; Bourhis, L. J.; Gildea, R. J.; Howard, J. A. K.; Puschmann, H. OLEX2: a complete structure solution, refinement and analysis program. J. Appl. Crystallogr. 2009, 42, 339.

[9] Spek, A. L. Structure validation in chemical crystallography. Acta Crystallogr., Sect. D: Biol. Crystallogr. 2009, D65, 148.

[10] Sheldrick, G. M. SADABS; Area Detector Absorption Correction; University of Göttingen; Göttingen, Germany, **2001**.
